# A Realist Synthesis of Literature Informing Programme Theories for Well Child Care in Primary Health Systems of Developed Economies

**DOI:** 10.5334/ijic.4177

**Published:** 2019-07-24

**Authors:** Pankaj Garg, John Eastwood, Siaw-Teng Liaw

**Affiliations:** 1 Department of Community Paediatrics, Liverpool Hospital, Liverpool, NSW, AU; 2 Specialist Disability Health Team, South Western Sydney Local Health District, NSW, AU; 3 South Western Sydney Local Health District, NSW, AU; 4 Ingham Institute of Applied Medical Research, Liverpool, NSW, AU; 5 School of Women’s and Children’s Health, University of New South Wales (UNSW), AU; 6 School of Public Health, University of Sydney, Sydney, NSW, AU; 7 School of Public Health, Griffith University, Gold Coast, QLD, AU; 8 Department of Community Paediatrics, Sydney Local Health District, Croydon, NSW, AU; 9 School of Public Health and Community Medicine, UNSW, AU

**Keywords:** Well child Care, theories, realist synthesis, integrated care

## Abstract

**Introduction:**

Well-child Care is the provision of preventative health care services for children and their families. The approach, however, to the universal provision of those services is contentious.

**Methods:**

We undertook a realist synthesis to enhance understanding of the theoretical mechanisms driving Well-child Care by searching for published and grey literature from multiple databases.

**Findings:**

Well-child Care is re-conceptualised as an integrated program delivered in the continuum of pregnancy, infancy and childhood. Depending on the context, Well-child Care can be a policy, a strategy, or an actual clinical practice that promotes child and family health. The main mechanisms include: role, training and continuity of health providers; administrators’ views of the return of investment on achieved outcomes; access to services by families; and the adaptation of programs to meet the dynamic needs of stakeholders. Evidence indicates that for most outcomes, Well-child Care is best delivered in partnerships between community health, social care, and early childhood education sectors.

**Conclusions:**

We conclude that Well-child Care policy and program leaders should shift their focus to the integration of: human and physical resources; policy instruments; and shared agreement on outcomes measures across health, social and education sectors. In addition, countries should work towards strengthening universal early education programs and parents’ health literacy regarding child development, health and safety.

## Introduction

Well-child Care refers to the provision of preventative primary healthcare services for children and their families. Different countries use, however, different terminology to describe the delivery of preventative health care services for children. These include, for example, child health promotion programs in the United Kingdom, child health surveillance programs in European Union countries, and Well-child Care in North America. As a significant proportion of the literature on preventive programs emanates from North America, we have consistently used here the term Well-Child Care. While there are differences in the use of the terminology in these programs, there are certain key components that are consistent, including: health supervision, anticipatory guidance, developmental surveillance, child and family psychosocial assessment, care co-ordination, immunisation, physical examination, and specific screening activities [1]. It is further conceptualised in the literature as a sub-component of primary health care for children that includes: Well-child Care, acute and chronic care for childhood health conditions, and co-ordination and follow-up for developmental problems. Thus, Well-child Care incorporates any program that targets child health promotion and child health surveillance activities with focus on prevention and early identification of problems.

Currently there is no international consensus on how Well-child Care activities are achieved within primary health sectors of developed countries. There are international differences in the provision of Well-child Care based on structural and practice level variables that are best reported using the conceptual framework of Starfield, which would place Well-child Care within the provision of outpatient and outreach clinical care, and within family and community services as defined in the World Health Organisation (WHO) continuum of care framework for women’s and children health [2,3]. Those framework looks at four structural level variables; (a) “regulation and governance” (i.e. the degree of the national organisation of Well-child Care and geographic distribution of health providers); (b) “financing” (the relative use of public and private finances for programs); (c) “health care professional” (training type and level of professionals); and (d) “accessibility” (financial, geographic, and other logistic flexibility for access to services). The practice level characteristics include: (a) “first contact with the family” (the type and role of health professionals as a first point of contact for parents); (b) “Coordination” (the degree to which care for acute and chronic conditions is provided in the same location and by the same provider of Well-child Care; (c) “Comprehensiveness” (extent to which all elements of Well-child Care are provided within a program that includes developmental, socio-emotional, educational, and social issues); (d) “Longitudinality” (the extent to which children and families see the same provider within the same setting over time; and (e) “Family-centred” (extent to which Well-child Care addresses the family and social context).

Previous research into Well-child Care has demonstrated a range of health-system and user benefits, including improved equity in access to specialist health services, reduction in avoidable hospitalisations [4,5], improvement in parent’s knowledge regarding normal child development and earlier identification for children with developmental delays [6].

Delivery of Well-child Care has remained a challenge as there are several user (parents) and provider (health system) related barriers to its delivery [1]. Despite a well-defined Well-child Care program in most developed economies, children from North America, the United Kingdom, Australia, New Zealand, Canada and European nations continue to commence primary-level education with unrecognised developmental vulnerability [7]. Alternative approaches to the provision of Well-child Care are therefore being explored that include the involvement of non-physician providers such as community health and peer support workers with the concurrent use of online digital platforms [8].

Well-child Care can be conceptualised as a complex organisational and social intervention delivered in open systems. In order to gain a sophisticated contextual understanding of its conception and delivery, there is a need to explain in which circumstances, why, to what extent, and for whom the programs of Well-child Care succeed or fail. Consequently, a more nuanced understanding of the “theoretical mechanisms” that drive the programs is required. Such a approach is important if we are to understand, for example, the decision making of program funders in relation to investment decisions for universal programs that are required to achieve the desired child and family health related outcomes. In Australia, for example, 1.34% of total health spending is invested on prevention. This is substantially less than many other comparable countries such as Canada, North America, United Kingdom and New Zealand [9].

Sayer [10], Pawson and Tilley [11] and others [12] have proposed employing a realist approach to analyse the delivery of such complex health and social interventions. An example of a previous realist synthesis is that undertaken by Molnar and colleagues who examined the role of unemployment insurance packages on health outcomes [13,14]. Such an approach seeks to postulate “theoretical mechanisms” within programmes that explain the observed processes and outcomes. Observational evidence alone cannot explain the causes of established uniformities between variables. It is necessary, therefore, to seek explanations as to why the certain relationships come about; and what it is that is “going on” in the system that connects its various inputs, outputs and outcomes [15]. In the context of a health program, mechanisms are not the program or service frameworks, but the response it triggers from stakeholders and resulting outcomes [16]. The analytical approach requires that the intervention resources to be taken into account, within the various contexts, and that the subsequent reasoning it triggers be assessed as possible mechanisms [15].

The realist synthesis reported here aims to understand the underlying “theoretical program mechanisms” that will explain the model and provision of Well-child Care.

## Theory and Methods

We will report our research findings according to the Realist And Meta-narrative Evidence Syntheses: Evolving Standards (RAMESES) [17]. Realist synthesis is utilised as it acknowledges the chain processes in health interventions that are influenced and modified by human and social factors. The stages of realist synthesis, as described by Pawson and colleagues [13] were utilised: (a) identifying the scope and research questions for review, (b) development of a theoretical conceptual framework, (c) literature search strategies and specified inclusion criteria, (d) critical appraisal of study quality, (e) use of an iterative process to gather further relevant data, (f) synthesis of data and refinement of theory; and (g) presentation and dissemination of findings. The main aim of the synthesis is to understand the context, interventions and mechanisms leading to perceived successes and failures of Well-child Care implementation [14].

Denise D’Souza [18] has elaborated on the context-mechanisms-outcome (CMO) configurations in realist synthesis. Context is conceptualised as a multi-faceted phenomenon that includes: (a) the pre-existing social conditions and context of the programs (context of action), (b) institutional structures, their material resources and provider practice, (c) agency, referring to the observable actions and reasons individuals engage in action or non-action; and (d) interpersonal relationships that have the potential of influencing the actions of others.

The term “mechanisms” refer to a hidden and inherent quality that operates within programs. They are sensitive to contextual variations and are responsible for the emergence of new initiatives and generation of outcomes [19]. Denyer and colleagues [20] suggest the additional distinction of interventions from mechanisms and propose the use of a context, intervention mechanism, outcome (CIMO) logic. This expanded model hypothesises that a change (O) occurs because of an intervention (I), operating on an underlying mechanism (M), in specific contexts (C) (Appendix Table 3).

### Evolution of research question and initial theory development

The background for this research question evolved over four years, from the authors’ clinical experience in community paediatrics, public health and general practice services. An initial narrative review of the literature shaped the theoretical conceptualisation of Well-child Care as a population-level program that negotiates interpersonal interactions within an institutional framework that is governed by state and national level policies (Appendix Fig 1).

Two research questions were informed by this realist review. We firstly sought to explain the explanatory and process factors underlying Well-child Care implementation, and secondly examine the effectiveness of current Well-child Care programs in achieving child and family related outcomes.

### Search strategy

A search of PubMed/PubMed Central, CINAHL, PsychInfo, SCOPUS, Cochrane Registry of Systematic Reviews and Google Scholar was conducted from 1963 to December 2017, with the following search terms: ‘Child Health Surveillance’ OR ‘Well-child Care’ OR ‘Child Health Promotion’. Relevant abstracts and titles were screened. We also identified reviews using a realist approach assessing preventive programs for child health [21,22,23,24,25].

An iterative search strategy was developed for identifying country-specific literature using publications by expert authors and websites of professional organisations and academic departments (Appendix Box 1). The search for articles stopped when “theoretical saturation” was achieved. That is when no new concepts on “theoretical mechanisms” emerged from the literature. Some literature was further reviewed following the recommendations of the editors and reviewers of the Journal.

The quality of Well-child Care programs was assessed using indicators gathered from child health surveys, longitudinal cohort studies, and international databases of leading organisations (Appendix Table 2).

#### Inclusion criteria

Studies, reviews and opinion pieces in English language that considered elaborating the contents, factors, cost-effectiveness, outcomes, and innovative approaches for Well-child Care for pre-school children originating from North America, Canada, New Zealand, Australia, the United Kingdom and European Union nations were included.

#### Exclusion criteria

Studies for school-aged children, youth preventative-health activities, hearing and newborn screening programs and articles with a specific focus on immunisations were excluded.

### Critical appraisal

Critical appraisal skills program (CASP) checklists were used for the evaluation of included studies [26]. Study quality was considered low if half or less than half of the criteria were met, medium if more than half but less than three-quarter criteria were met, and high if most were met. The modified Oxford Centre for Evidence tool was used to grade study level (Appendix Table 1). As some recently conducted systematic reviews have already evaluated the effectiveness of Well-child Care programs using independent author consensus, the utility of repeating this process for the purposes of this realist synthesis was considered unnecessary [27,28].

### Data analysis and synthesis

All abstracts, titles and relevant passages from included full-text studies were collated into word documents and managed using NVivo qualitative software [29]. A synthesis of qualitative data was conducted by taking into account the context, mechanism, intervention and outcome themes. Textual data was coded ‘line by line’ and ‘paragraph by paragraph’, employing an inductive approach described elsewhere [30]. The data synthesis focused on refining the initial theoretical framework (Appendix Fig 1). The main mechanisms that are a form of testable hypothesis, were developed using an abstract inductive and abductive approach, and are presented as *‘if’* and *‘then’* propositions [31].

## Results

**Figure 1:**
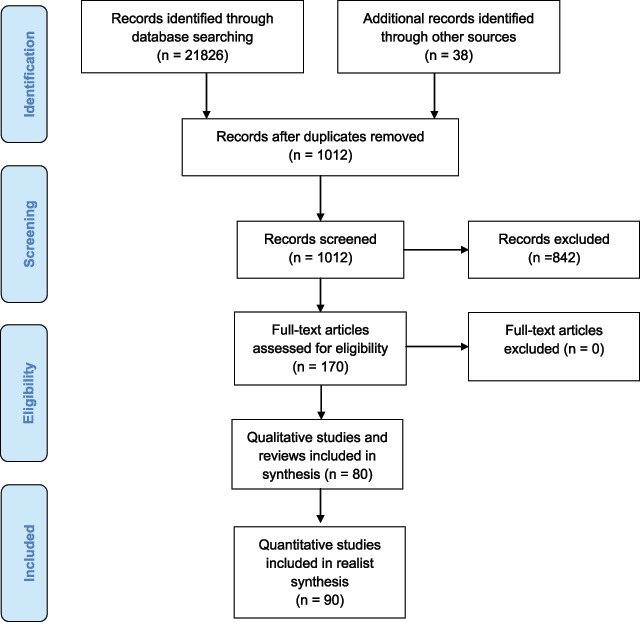
Flow Diagram of included studies.

Search results are highlighted in Figure 1. A total of 1012 non-duplicate abstracts were extracted from a total of 21826 citations. One-hundred and seventy references were evaluated in detail. Ten documents were also reviewed subsequent to the initial draft, on the suggestions provided by the Journal. Eighty-three of the identified studies focused on understanding factors that impact the coverage of Well-child Care programs. A further 49 literature reviews focused on cross-country comparisons of Well-child Care and health promotion and intervention programs for promoting early childhood development. The remaining 38 documents varied from country level frameworks for universal access to child and family services through to evaluation of specific programs for vulnerable families.

### Contexts and interventions for Well-child Care

Well-child Care is re-contextualised as a strategy, policy and clinical practice by various agencies and sectors to promote child health at primary health care level. Table 1 elaborates the various interventions that promote Well-child Care. These are delivered in the format of policy, programs, and strategies by health, family and community services, non-governmental organisations, local government agencies and educational sector.

**Table 1:** The CIMO configuration of Universal and Targeted Well Child Care*.

Context	Interventions	Mechanisms	Outcomes

Universal Well-child Care	Various programs, e.g. Families NSW in NSW, Best start in Victoria, Australian Medicare Healthy Kids Check (July 2008 to July 2016)	Evidence for effectiveness	Variability in delivery-based on context and activated mechanism
Well-child Care is important as early childhood period is critical	North American Bright Futures program	Training and role of the staff (GPs, paediatrician, nurse, changing workforce)	Delays in identification of children with DD
There is either a national consensus on Well-child Care or no consensus	New Zealand Well-child Care program	Funding mechanisms	Improvement in parents knowledge
	Personal Health Records (PHRs)-contents	Best approach -Screening, surveillance or health promotion	Reduction in avoidable hospitalisations
	Guidelines for Well-child Care	Parenting skills (health literacy of parents)	Identification of parental vulnerabilities
	Screening programs (oral screening, STEPS, hearing screen)	Population characteristics	Unmet parenting needs
		How do parents and providers use PHRs	Parents satisfaction with the programs
		Communication style- reassurance and partnership	Little information sharing between Well-child Care providers
Children in vulnerable populations are at risk for poor outcomes and neglect, and access less health visits	Specific programs for vulnerable populations	Social determinants –isolation, poverty, unemployment, mental health issues	Success in maintaining safety and well-being of children
	Sustained nurse visiting program	Feeling disempowered	Inconsistent engagement of vulnerable families
	Tiered approach for identification of vulnerable families	Perception of families regarding first contact with health provider	Missed opportunities at immunisation visits
		Partnership-non-judgmental style	Integration between services remain limited
		Provider-task-oriented	

* CIMO–Context-Intervention-Mechanism-Outcomes, NSW – New South Wales (Australia), STEPS–State-wide Eyesight Pre-schooler Screening, DD–Developmental Disability.

Two broad contexts for Well-child Care emerged. Firstly, of “proportionate universalism” and preventive child care for whole populations; and secondly a targeted approach for vulnerable populations. Most developed economies have invested in the development of robust access to primary health services that includes universal access to services and programs that encourage preventive care for children and families. The context-intervention-mechanisms-outcomes configurations for these two contexts are elaborated upon in Table 1. Proportionate universalism relates to the provision of a suite of services for all children and families with the provision of additional support commensurate with additional needs. Alternatively, targeted approaches are delivered specifically to vulnerable populations that are identified using pre-defined criteria.

Many states in North America such as Florida, Vermont, Georgia, Oklahoma, New Jersey, Illinois, Iowa, New York, and West Virginia also have policies promoting universal access to preschools. Oklahoma was one of the first states in North America to offer universal preschool for 4-year-olds. Evaluations showed that students enrolled in Oklahoma’s pre-kindergarten program consistently outperformed those not enrolled in the program, and this effect was observed across racial groups and in low-income and middle-class families [32]. There are, however, several disparities in equity and access to state funded preschool programs in America [33].

#### Explanatory factors and interventions for Well-child Care

A realist approach identified common contextual themes including the importance of the early childhood period and Well-child Care. Implementation challenges included what to deliver, for whom, and how. These along with challenges in measuring outcomes for Well-child Care programs, acted as the main mechanisms for the continuation or discontinuation and further development of programs. Opinion leaders and researchers successfully argued, in most developed economies, to sell the provision of universal Well-child Care to policy makers, and programs subsequently evolved based on the role of health providers, and evidence for the various components of Well-child Care [34]. They also highlighted, however, the challenges in delivery, and called for further research on outcomes emerging directly from the programs. While in the UK the program moved away from an active child health surveillance program to a child health promotion program, North America emphasised clinical practice redesign and non-medical models of service delivery [8]. In Australia the programs are particularly focused on socially disadvantaged populations and enhancing the role of nurses in general practice [35]. Sweden, Denmark and the Netherlands have achieved high preventive coverage but these countries also face challenges in adapting the programs to current demographic changes [36].

The variability of the universal Well-child Care programs was explained by structural and organisational factors. In Australia, for example, there is no national consensus on the provision of Well-child Care. In particular there is no policy on how general practitioners (GPs) can systematically deliver preventative health checks [34]. A primary care routine health check at 4 years was funded for GPs in Australia from July 2008 to July 2015 for identification of health and developmental problems prior to school entry but was discontinued due to a lack of effectiveness [37]. In North America the responsibility for Well-child Care provision rests with primary care paediatricians, and there are comprehensive developmental screening and surveillance guidelines in the Bright Futures program endorsed by the American Academy of Paediatrics [38]. Despite this, less than half of American pre-school children are reported to have a standardised developmental screening measure and many parents report unmet needs regarding health promotion advice for their children [39].

A review by Public Health in England supported the evidence for the United Kingdom’s multicomponent Healthy Child Programme [40]. The New Zealand Well-child Care program (Tamariki Ora) is provided by a GP, child and family nurse or social worker. The contents of the program are evolving to meet the variability in coverage and referral rates across locations, providers and ethnic groups [41].

In the European Union the provision of Well-child Care is mostly by GPs but paediatricians and a combined system of GPs and nurses also play a major role. In Sweden there is a chain-of-care arrangement, while in the Netherlands there is a trans-mural model to facilitate better integration and coordination of child health services [42].

All developed economies have formulated specific programs for disadvantaged populations in a bid to reduce health inequalities with a focus on early childhood education, mitigating psychosocial stressors, responsive parenting programs and early identification of developmental and behavioural problems in children (Appendix Box 1). Many of these programs include a component of sustained nurse home visiting for provision of support to vulnerable families [43].

### Mechanisms explaining variability of Well-child Care

Seven main theoretical mechanisms that affect the provision of Well-child Care either directly or indirectly are presented as ‘*if*’ and ‘*then*’ propositions.

#### Conflicting or low quality evidence for outcomes

These propositions state that:

- *If evidence for a component of Well-child Care is lacking, then it is challenging for policy makers to invest more money for supporting structured Well-child Care activities in primary health services*.
- *If there is uncertainty in demonstrating which child health and family level outcomes is the direct result of Well-child Care provision, then that specific component of Well-child Care program is at threat of discontinuation*.

These two propositions are supported from systematic reviews that conclude that the quality of evidence is sub-optimal to inform a change in practice. This means that policy makers are less likely to prioritise Well-child Care [27,28]. Oberklaid has documented these challenges for policy makers in response to the rescindment of a four year-old Healthy Kids Check in Australia because of the mounting evidence that it was not meeting child health outcomes [27].

Heckman has presented compelling arguments for policy makers to shift away from focusing on only academic outcomes and school readiness as a measure of success of early childhood programs [44,45]. He has demonstrated several positive long term outcomes at 30 years of age for children enrolled from 8 weeks of age to five years in the Carolina Abecedarian Project (ABC) and the Carolina Approach to Responsive Education (CARE) programs. An overall 13% “Return on Investments” was demonstrated with positive outcomes on long term measures such as overall health, occupational and social success (e.g. participants completed high school, more likely to be employed and less likely to have a criminal record) [46,47].

#### Provider-parent interactions

This proposition states that:

*If parents and health professionals achieve concordance of topics to cover during a routine health visit, then more age appropriate Well-child Care activities are provided*.

This is supported by literature that demonstrates greater consensus between parents and physicians on topics relating to Well-child Care, when a family-centred approach is employed. In a survey of 137 parents and 31 physicians, a targeted method of anticipatory guidance resulted in a greater provision of Well-child Care [48].

#### Health systems for Well-child Care

This proposition states that:

- If practice nurses and health visitors are involved in Well-child Care, then more education is provided regarding Well-child Care topics; and
- *If the health systems promote continuity of care, with increased time and organisational supports to health providers, more Well-child Care activities will be done*.

Practice nurses, health visitors, nurses and other non-physician providers have been shown to be more effective for parent education, anticipatory guidance and early identification of developmental-behavioural issues [49].

Continuity of care is a well-studied health system factor, that is often associated with improved parental satisfaction and increased availability of the provider [50]. Continuity of care, however, is often challenging to achieve even for highly-developed health systems. For example, in Australian Capital Territory, only 39% of GPs were noted to provide continuity of care over a period of eight years [51]. Another example is the, Montefiore Medical Center in New York City in North America that has developed a comprehensive Integrated Care Delivery for Vulnerable Populations [52]. Several strategies have been used to promote access for families and children for primary health care needs, such as expanding hours in the evenings and weekends, drop in clinics and integration of electronic medical records across various hospital and community systems. This has resulted in improved outcomes for families and children with developmental and behavioural problems [53].

#### Access to health care

This proposition states that:

If families can access routine health checks consistently, then more Well-child Care activities will be offered, resulting in better child and family level outcomes.

Access for preventative health checks vary by social class and insurance status. Uninsured children from immigrant families receive less than the recommended Well-child Care visits, while married first-time mothers have been shown to adhere to the recommended schedule of visits [39,54]. Flexible work options for parents is often associated with higher preventative health care, given the mitigation of a number of access issues including unexpected time off work, other childcare arrangements and ability to more assertively manage crisis [55].

In addition digital technology approaches are increasingly being used to improve access to Well-Child Care including: telemedicine, and parent coach led models [8].

#### Policies and national framework

This proposition states that:

- If there is a national framework for Well-child Care program, then the population level coverage of Well-child Care programs are enhanced; and
- *If there are robust policies within health, social and educational sectors, then there is an increased provision of Well-child Care*.

Sweden is a success story with a robust national Well-child Care program complemented by a supportive early childhood education framework [56]. This has resulted in high population coverage of Well-child Care programs with high enrolment rates in the early education sector.

In the North American states of Kentucky and Idaho, policy changes such as incentive payments for physicians and waiving insurance premiums for families, enhanced the preventative care usage by families [57]. Cheeseman has argued, citing the example of the Australian Early Childhood Policy Initiative, that an enhanced focus for overcoming social disadvantage often comes at the expense of promotion of universal rights for a comprehensive early childhood education experience [58]. It is important to note here, that although it was previously easier to promote politically, in North America and European Union countries, universal access to early childhood programs, the increasing inequity in health and social outcomes has recently shifted the focus to vulnerable populations [52].

#### Diversity and changing epidemiology

This proposition states that:

*If there are changes in population demography, political philosophies or epidemiological patterns of childhood presentations in primary health care, then policy makers and staff involved in the provision of Well-child Care programs have to tailor programs accordingly*.

There are rapid changes in the demography of populations in developed economies that has been driven by adapting immigration policies and internal political changes [59]. Coinciding with these societal shifts there is a well-documented increase in neurodevelopmental and behavioural problems in paediatric practice [60]. Along with this there is a contribution of poverty and exposure to adverse childhood experiences on children’s development and behaviour. Adverse childhood exposures such as intimate partner violence, parental mental health issues, illicit substance abuse and alcohol, and families fleeing warn torn regions are well-documented to result in short and long-term health and developmental outcomes [61].

#### Inter-sectoral collaboration and integration

This final proposition states that:

*If there is integration and collaboration between health, education and social sectors, there is an increased likelihood of success for Well-child Care programs*.

Building on our initial framework, Well-child Care emerges as a multi-component concept that cannot be delivered through the health sector alone. There is a need for inter-sectoral integration between social, health, education and non-governmental organisations. Programs have to address the varied needs of stakeholders and evolve with the efforts of local level leadership. The various components of the integrated Well-child Care framework are shown in Figure 1. This Well-child Care model is supported by the successful incorporation of inter-sectoral actors in the Swedish Well-child Care program for improving parental education, and links well with the World Health organisation framework on integrated people-centred health services [62,63]. Similarly, Coker has argued that community connections and a one-stop shop model is more likely to address the major drivers underlying adult diseases such as poverty, unemployment and risk taking behaviours [64].

## Outcomes: the empirical evidence base for Well-child Care

**Figure 2:**
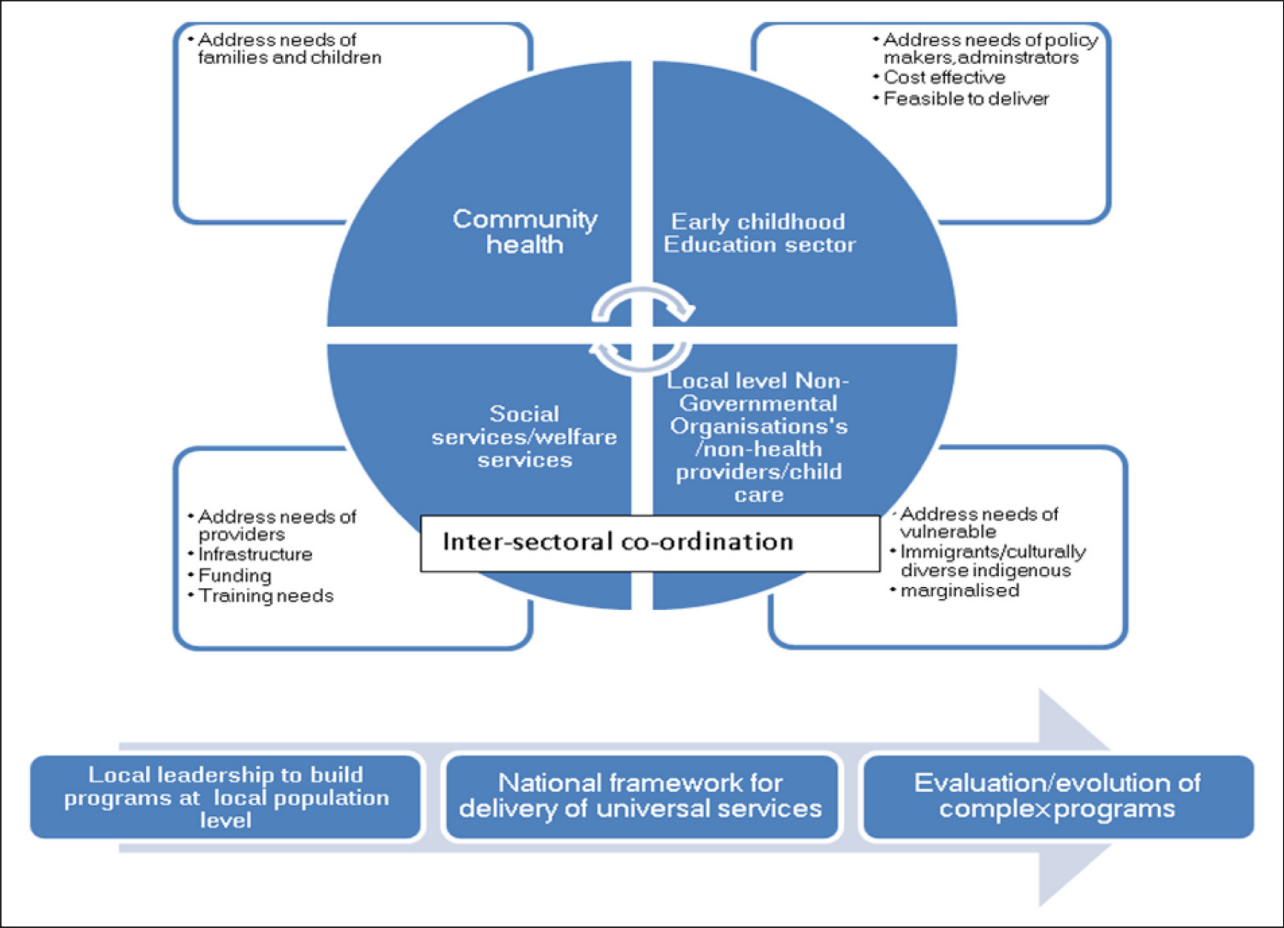
The components for Well Child Care using the WHO Integrated Care Models framework.

The synthesis of the evidence demonstrating the effectiveness of Well-child Care is presented below that support the integrated model of Well-child Care highlighted in Figure 2. Tables 4 and 5 in the Appendix summarises the main empirical evidence that support the concept of integration of services for Well-child Care.

### Parental coaching programs – Integration between community health, social welfare and non-governmental organisations

The parenting programs for promoting child health are delivered by a variety of health professionals with background in social work, psychology, education, nursing and psychiatry and often in partnership with social services, non-governmental organisations and community health. A meta-analysis of 50 intervention studies delivered by diverse providers and professionals, of the Incredible Years Parent Intervention (IYPT) program with 2472 and 2273 participants in intervention and control groups, has demonstrated benefits with a mean effect size of Cohen d = .27 for improvements in disruptive behaviours in children [65]. Similarly meta-analysis of Triple P parenting programs involving 11,797 families in 55 studies showed an effect size of 0.38 for parenting, 0.35 for improvements in common childhood problems, and 0.17 for improvements in parental well-being [66]. Another meta-analysis of 77 studies with relevant comparison groups (45 of them being randomised controlled trials) studied the effectiveness of the various components of parent training program; showed overall weighted effect size across all outcomes to be 0.34 (95% CI 0.29 to 0.39). Program components with significantly larger effects on child’s behavior, included positive interactions with the child (regression weight 0.284), time out (regression weight 0.170), consistent responding (regression weight 0.333) and practice with the child (regression weight 0.234) [67].

### Developmental surveillance programs –integration between public community health services, general practices, and early childhood sector

Developmental screening, counselling and referrals at preschools has been demonstrated to improve the rates of referrals for further assessment of developmental problems [68]. Developmental screening and surveillance are core features of the American Academy of Paediatrics Bright Futures program [38]. The poor uptake, however, of screening tools for developmental surveillance by health professionals globally, highlights the need for an integrated approach between early childhood education, community health and general practice sectors [69]. Glascoe’s work on identification of developmental problems by doctors, and how it links to the educational services in the community needs more analysis [70]. Systems to screen and evaluate for child development and behaviour and family stressors do exist, and need consistent implementation [71].

Further, non-face to face approaches (two Randomised Controlled Trials) using internet based peer support and coach and inclusion of non-medical developmentally-trained professionals has been shown to enhance the provision of age appropriate anticipatory guidance for families [8].

Similarly, a meta-analysis by Downing and colleagues, demonstrated that behaviour change interventions in a preschool, day care, and community settings (sample size varying from 22 to 885), resulted in positive health promoting behaviours [Mean reduction, –17.12 (95% CI –28.8 to –5.40); –18.91 (95% CI –33.3 to –4.50, respectively] [72]. The role of early childhood educators for developmental screening and surveillance is also well-documented and provides further evidence for integration of early childhood and primary health care sectors [73].

### Integration between community Health, Social services and paediatric health services

Parental education programs for injury prevention delivered by community health nurses during home visits, or by paediatricians or general practitioners at their practices have shown a 18% mean risk reduction (95% CI 5–29%) in injuries in the intervention arm [number of participants varied from 47 to 348] [74]. Similarly systematic screening for psychosocial problems improved referral rates for intervention programs (70% versus 8%; adjusted odds ratio [aOR] = 29.6; 95% confidence interval [CI], 14.7–59.6), and linkages of families with community resources (39% vs 24%; aOR = 2.1; 95% CI, 1.2–3.7) [75]. Similar successes have also been obtained when social work, nursing, and medical teams work together for developing interventions for achieving improvements in rates of obesity and overweight [76].

Several programs for vulnerable families, such as the North American Healthy Steps for Young Children, which is a collaboration between paediatric primary health staff, health specialists from diverse professional backgrounds, and a number of cooperating local foundations, has been shown to enhance Well-child Care proportionate to the needs of the families [77].

### Primary Health system practice level factors enhancing Well-child Care

Several studies have demonstrated key evidence on practice level factors that improve access and engagement of families with Well-child Care activities. Open access scheduling has been shown to reduces missed appointments from 21% to 9% and improves immunisation rates from 59% to 74% for a vulnerable population groups [78].

Practice-based education, using the components of audit, identification, and implementation of new processes improved age-appropriate anticipatory guidance rates from 2.2% (95% CI 0.8–5.9) to 18% (95% CI 10.3–29.9), but demonstrated no change in parenting knowledge [79].

The use of a family-centred approach embedded within a routine Well-child Care program significantly improves earlier identification of social-emotional problems in children (OR 1.44 (0.96; 2.18), Phi = .03) [80].

Table 2 provides a framework for the various levels and degree of integration and the policies that will be required at states, provincial and national level for integration of services for Well-child Care [81].

**Table 2:** Components of Integrated Model of Well child Care.*

Component	Sectors	Type of Integration	Common outcomes measures

**Pregnancy** Early identification of psychosocial stressors-domestic violence, depression	***Social sector*** – reporting systems for family vulnerabilities, interventions ***Health sector*** – nursing and medical teams, private and public sectors ***Non-governmental organisations*** Programs for pregnant women ***Local Level Government*** Educational programs, parenting groups	**Organisation** – formal memorandum of understandings, development of information sharing platforms with respect to personal privacy **Service Integration** – joint programs for vulnerable population groups, multidisciplinary teams from various organisations **Clinical Integration** – shared guidelines and protocols	Proportion of babies’ breast fed up to 6 months exclusive (%) Proportion of mothers identified with postpartum depression Proportion of boys (<20 yrs.) identified overweight + and obese Proportion of children with developmental vulnerabilities in at least one domain at school entry
**Postnatal, Infancy, toddlerhood and early childhood** Age appropriate anticipatory guidance on sleep, feeding, discipline, safety, developmental milestones-improving health literacy of families Screening and Surveillance for developmental-behavioural problems for early identification, referral and linkage to intervention programs Screening for hearing and vision Monitoring of Physical growth Psychosocial assessment for parental issues-jobs/illnesses in the family Care-coordination	***Community and Social services*** *Health services* – primary and secondary levels of care, specific risk groups such as preterm follow up programs, community health, nursing and family and general practice teams, format of well child care checks ***Early childhood education*** Developmental surveillance and screening for early identification of developmental problems ***Local Level Government*** – councils, educational, and health promotion activities at libraries, and other community programs	**Organisation** – formal memorandum of understandings, development of information sharing platforms with respect to personal privacy **Service Integration** – joint programs for vulnerable population groups multidisciplinary teams from various organisations **Clinical Integration** – shared guidelines and protocols	Proportion of babies immunized fully 12 to 23 months Continuity of provider for well child care (usual source primary care provider) Proportion of children with ASD (2–17 years), most recent estimates Proportion watching TV more than 1 hr and less than 4 hrs (1–17 years) Developmental screening completed (10 months-5years) Family involved in home visitation program Proportion of children <5 yrs visiting dental worker Proportion of 4 year old children enrolled in an early childhood program Annual number of deaths and injuries 1–14 yrs. per 100000(1991–1995). Child maltreatment deaths per 100000 children (up to 15 yrs)

* As highlighted in table above, both horizontal and vertical integration, at micro-level for individuals, meso-level for specific populations, and macro level for whole populations will be needed.

## Discussion

The theoretical propositions highlighted in this paper enhance our understanding of complex mechanisms that affect child health promotion and child health surveillance activities, the two core activities of Well-child Care. The empirical evidence on effectiveness of Well-child Care elaborated in this paper highlight that outcomes for Well-child Care are better when there is some level and degree of collaboration between nursing, medical teams, education, social care and non-governmental organisations. This is because the developmental domains of physical, language, attachment, socio-emotional and cognitive wellbeing are achieved by children across five different stages of pregnancy, postnatal, infancy, toddlerhood and early childhood. Thus, each domain and each stage require specific supports and skills highlighting the inter-disciplinary nature of Well-child Care, and need for integrated model of care (Table 2). This interdisciplinary Well-child Care model of development is informed by the World Health Organisation Reproductive Maternal Newborn Child Health continuum framework, and is increasing relevant with the current patterns of migration of communities driven from economic reasons, war-torn countries, climate changes and the increase in the inequity of access to services among these communities in developed economies [3]. Ingrid et al, has also elaborated in their realist synthesis various “mechanisms” in social paediatrics that identify “how” the outcomes are achieved for vulnerable communities [82]. This include: (a) shared values and willingness of partners to share status and power that result in horizontal partnerships, (b) by building trust of vulnerable communities that result in greater acceptance of care, (c) Institutional knowledge support enhances practitioner’s confidence that results in increase of client referrals to needed services, and (d) empowering vulnerable communities result in increase in service utilisation.

The synthesis of findings reported here will assist policy makers, academics and researchers when making due considerations for the development and adaptation of existing Well-child Care programs into multi-component integrated programs. The findings are also likely to be transferable to low and middle income settings such as China, India and Brazil that are working towards strengthening their primary health care systems [83].

Previous reviews of child health programs in selected developed countries (Australia, Canada, North America and Sweden) have documented the cross-country variability in specific components of Well-child Care, and our review provides a comprehensive explanation for these variations [84]. The variations in Well-child Care programs have called for a partnership between multiple European organisations and countries for mapping and appraising various models of child health (MOCHA project) [85]. The focus of this collaboration is on the specific format, and contents of health checks by medical providers, and not so much on the integration of the Well-child Care components.

An integrated model of Well-child Care for the most vulnerable population in a defined Sydney region is currently being evaluated [86]. The components of this program include: care coordination, place-based collaboration in most disadvantaged local government areas, general practice engagement, capacity building and linkage for family health improvement, and system partnerships. This is being achieved through formalised partnerships and memorandum of understandings between education, juvenile justice, health, substance abuse, mental health, legal, and housing and community services [86].

Models of integrated child care, including the above Sydney initiative, are increasingly taking into account both horizontal and vertical level integration approaches [87]. The Queensland government in Australia has developed an integrated approach for tackling childhood overweight and obesity in the state by targeting prevention and early intervention within primary healthcare and integration of these services with secondary, tertiary, and quaternary level services [88]. This is led by a Children’s department within the state government. Similarly in the United Kingdom the creation of Imperial Child Health general practice hubs allows for provision of paediatric specialists in out-of-hospital settings [89]. Such integrated models of care delivery require a cultural shift in the training of doctors, nurses, and other providers, and willingness to share power and status. Success of such programs will be determined by a common cause, vision, strategy, joint funding, planning and service delivery and evaluation and quality improvement process [87].

There are a number of other integrated health care initiatives in the European Union that are evolving from collaborative research partnerships towards the development of integrated care frameworks [90]. An European example of a successful integrated care initiative is in the Basque county of Spain. The levers of change for this program have included the strategic political decision at a local government (state) level to move on the message of collaboration. This resulted in the vertical integration of hospital and health centres and formation of 13 Integrated Care organisations and 3 mental care networks. This was achieved by merging of all hospitals and community health services (a total of 21 entities) [91]. Similarly other local integrated care initiatives evolved from local health and social leaders driving a roadmap of local government integrated initiatives.

The integrated Well-child Care model presented in Figure 2 and Table 2, takes into consideration family and job level factors such as the impact of maternal leave allowances on enhanced preventative child healthcare visits. These are very important considerations as such social level variables have been demonstrated to affect national level child health indicators in countries with different political traditions [92].

Integrated programs utilising innovative Well-child Care models have been shown to be cost-effective for obesity and injury-prevention education programs for managed health organisations in North America [93,94,95]. Further research into the cost-effectiveness of multi-sectoral Well-child Care in different health and organisation settings are required.

## Conclusions

We found in the literature uncertainty regarding the best model of Well-child Care for achieving desirable child and family health outcomes. This paper bridges the gap by explicitly highlighting the need of an integrated framework for health, social welfare and education sectors to work synchronously to provide Well-child Care activities and to achieve measuring population level child health and wellbeing outcomes. There is sufficient empirical evidence for the benefit of multi-component programs on preventive activities for children. Guidelines for Well-child Care for professionals should be simple and meet the needs of the providers and families for them to be acceptable to all stakeholders. Family-centred care, promoting health literacy, enrolment of pre-schoolers in quality education programs, reduction of access barriers through innovative technological approaches, enhancing inter-sectoral coordination, continuity of care and supporting primary health providers are best practice elements in Well-child Care. There is a need for development of coherent integrated outcome measures for inter-sectoral collaboration between health, social welfare and education services that are measurable and meets the service and budgetary objectives of diverse service providers.

## Additional File

The additional file for this article can be found as follows:

10.5334/ijic.4177.s1 Appendix. The Appendix contains details of literature search, supporting tables and list of references included in the realist synthesis.
