# Supplementary material for: A Realist Synthesis of Literature Informing Programme Theories for Well Child Care in Primary Health Systems of Developed Economies
Source: Int J Integr Care. 2019 Jul 24;19(3):5. doi: 10.5334/ijic.4177 (PMC6659757; doi:10.5334/ijic.4177)
Supplement: Appendix. — The Appendix contains details of literature search, supporting tables and list of references included in the realist synthesis. [file ijic-19-3-4177-s1.pdf]

Fig 1. Initial theoretical framework of complex interplay of factors for delivery of Well Child Care

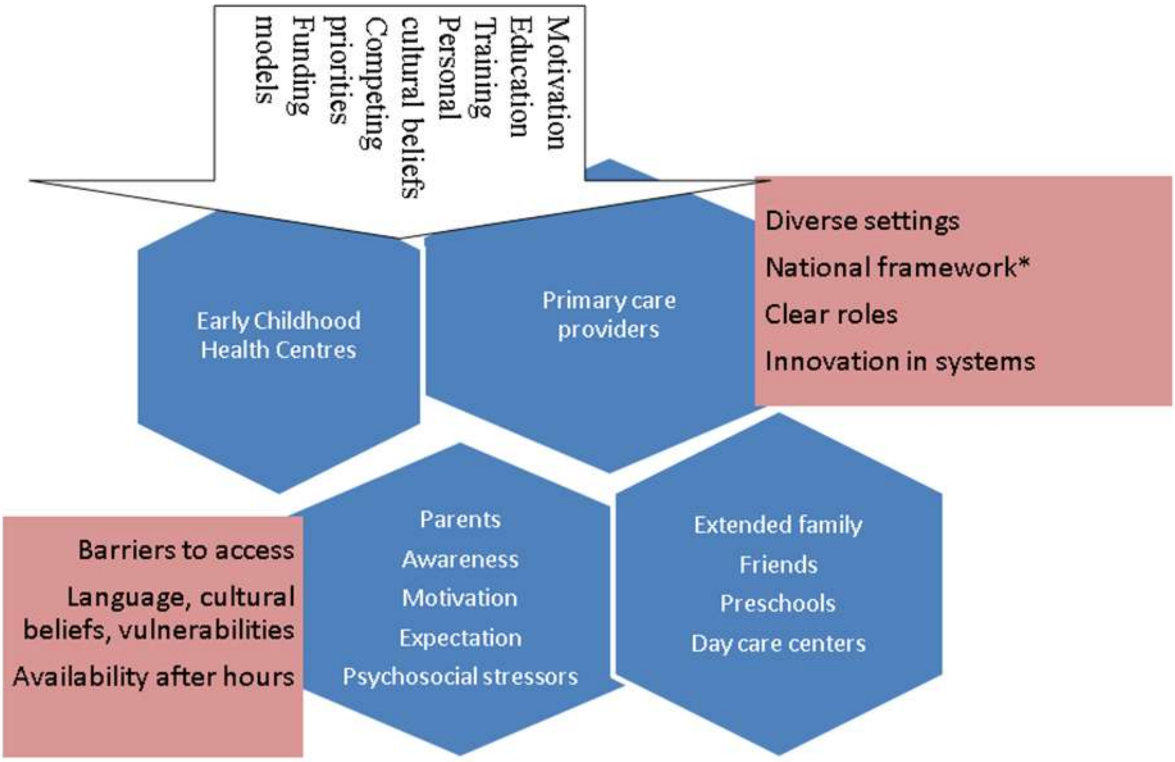

Box 1

Iterative search of databases for following authors

[Mitch Blair and David Hall (UK), Paul Dworkin and David Bergman (USA), Frank Oberklaid, Sharon Goldfield, Helena Britt (Australia), R Kornfält, Sven Bremberg and Robert Kristiansson (Sweden), Magdalena Janus (Canada), John Eastwood (New Zealand), AM Skovgaard, EM Olsen and Sondergaard G (Denmark), Sijmen Reijneveld (Netherlands), Diego Van Esso (Spain), Stefano Del Torso (Italy), Michael Rigby (European Union)].

Main WCC programs for vulnerable families

Family Start (New Zealand), Sure Start (UK), Every Child matters (UK), First Duty Toronto (Canada), Healthy Child Manitoba (Canada), Head Start & Early Head Start (USA), First 5 California (USA), Stronger Families and Communities (Australia), and Families NSW (NSW, Australia).

Fig 2. Country wise publications in SCOPUS database*

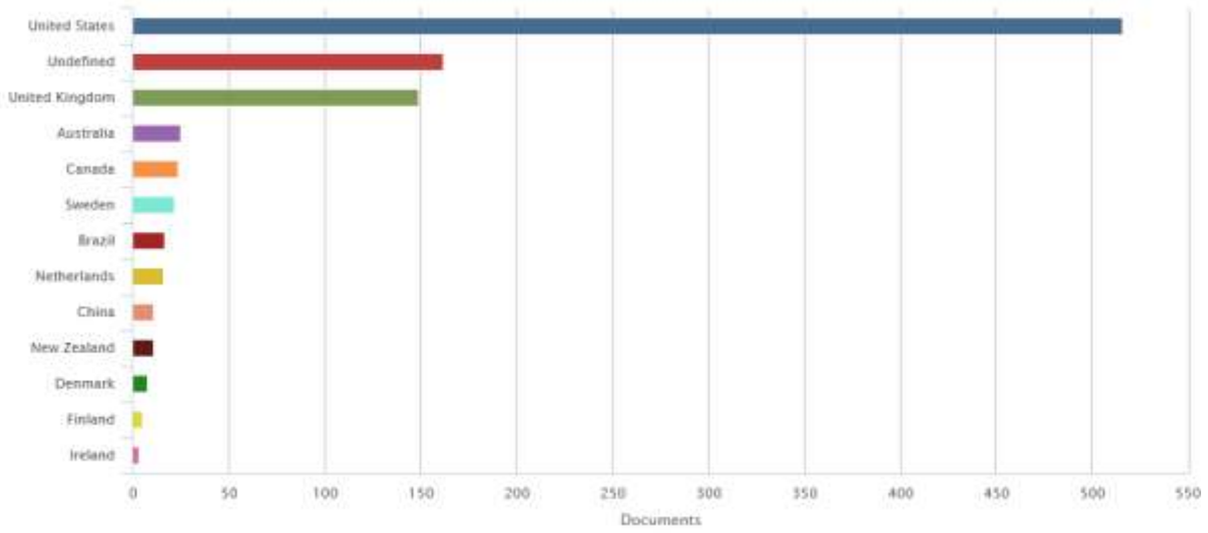

*Search strategy “Child health Surveillance” OR “Well Child Care” OR “Child health promotion” (1963- 2017)

Table 1. Levels of evidence based on Oxford Evidence Based Medicine used in the study

Level	Description
Level I	Evidence from Systematic Reviews of RCTs or meta-analysis of RCTs or ≥3 good quality RCTs
Level II	One well designed RCT
Level III	Quasi- experimental well designed controlled trial, no randomization, quality case-controlled studies or large cohort studies
Level IV	Systematic review of descriptive and qualitative study
Level V	Opinion pieces and expert opinions

Table 2. References used for outcomes and indicators of quality of WCC programs

Country	Data source
USA	"The Timing of Maternal Depressive Symptoms and Mothers' Parenting Practices with Young Children: Implications for Pediatric Practice," Kathryn Taaffe McLearn<br><br>National Survey for Child Health 2011-2012,National Survey of Early Childhood Health,Centre for Disease control<br><br>Commonwealth fund<br><br>National Health and Nutrition Examination Survey 2011-2012<br><br>http://www.ada.org/~/media/ADA/Science%20and%20Research/HPI/Files/HPIBrief_1015_1.pdf
Australia	Australian Early Development Index (AEDI)<br><br>Australian Health Survey (2011-2013), Longitudinal Study of Australian children (LSAC)<br><br>http://www.a4.org.au/prevalence2015<br><br>Screen time of infants in Sydney, Australia: a birth cohort study.<br><br>Meena Chandra, Bin Jalaludin, Susan Woolfenden, Joseph Descallar, Laura Nicholls, Cheryl Dissanayake, Katrina Williams, Elisabeth Murphy, Amelia Walter, John Eastwood1,Valsamma Eapen<br><br>The Watch Me Grow Study Group<br><br>https://www.aihw.gov.au/reports/dental-oral-health/oral-health-and-dental-care-in-australia-key-facts-and-figures-2015/contents/summary
Canada	National Survey of early Childhood health, Early Development Index

	http://www.cfhi-fcass.ca/Migrated/PDF/ResearchReports/CommissionedResearch/cr_contcare_e.pdf<br><br>https://www.canada.ca/en/public-health/services/diseases/autism-spectrum-disorder-asd/surveillance-autism-spectrum-disorder-asd.html
New Zealand	New Zealand Health Survey 2013/14<br><br>Thio et al. Postnatal depressive symptoms go largely untreated: a probability study in urban New Zealand Soc Psychiatry Psychiatr Epidemiol. 2006 Oct;41(10):814-8.<br><br>Growing Up In New Zealand Longitudinal study (GUiNZ)<br><br>National and DHB Immunisation data (Ministry of Health, New Zealand)<br><br>N Z Med J. 2011 Feb 11;124(1329):16-25.Continuity of care with general practitioners in New Zealand: results from SoFIE-Primary Care. Jatrana S1, Crampton P, Richardson K
Sweden	Acta Psychiatr Scand. 1997 Jan;95(1):62-6.Screening for postnatal depression in a population-based Swedish sample.<br><br>Wickberg B[1], Hwang CP.<br><br>Survey of the pre-school child health surveillance programme in<br><br>Sweden R Kornfa¨lt (Acta Paediatr)<br><br>UNICEF/WHO estimates for immunisation coverage<br><br>Acta Paediatr. 2009 Dec;98(12):1956-8. Is the prevalence of overweight and obesity declining among 4-year-old Swedish children? Bergström E[1], Blomquist HK.<br><br>J Epidemiol Community Health. 2003 Sep;57(9):724-9. Association between childhood community safety interventions

	and hospital injury records: a multilevel study.Sellström E[1], Guldbrandsson K, Bremberg S, Hjern A, Arnoldsson G.<br><br>Fernell E[1], Bremberg S. Lakartidningen. 1996 Jun 5;93(23):2237-9.<br><br>[Mild mental retardation is very seldom discovered at child health care centers. Diagnosed in every fifth child prior to the compulsory school attendance].<br><br>Association between continuity of care in Swedish primary care and emergency services utilisation: a population-based cross-sectional study<br><br>Hannes Kohnke,,Andrzej Zielinski, Scandinavian Journal of Primary Health Care, Volume 35, 2017 - Issue 2
United Kingdom	Public Health , England, http://fingertips.phe.org.uk/profile-group/mental-health/profile/perinatal-mental-health/data#page/0<br><br>Curtin et al. Determinants of vulnerability in early childhood development in Ireland: a cross-sectional study. Available: http://dx.doi.org/10.1136/bmjopen-2012-002387<br><br>ONS Longitudinal study, https://www.ons.gov.uk/aboutus/whatwedo/paidservices/longitudinalstudyls
Denmark	Transition from ECEC to school. https://www.oecd.org/edu/school/SS5-country-background-report-denmark.pdf<br><br>http://www.nordicwelfare.org/PageFiles/415/Inspirationsh%C3%A4ften/5ENG_LR.pdf<br><br>Copenhagen Child Cohort 2000 (CCC2000) studies<br><br>General Practice and Primary Health Care in Denmark, Kjeld Møller Pedersen, John Sahl Andersen, Jens Søndergaard,. Journal of the American Board of Family Medicine<br><br>https://www.oecd.org/health/health-systems/Primary-Care-Review-of-Denmark-OECD-report-December-2016.pdf
Netherlands	Acta Paediatr. 2005 Jul;94(7):935-42.<br><br>Infant milk feeding practices in the Netherlands and associated factors.

	**http://www.oecd.org/edu/EDUCATION%20POLICY%20OUTLOOK_NETHERLANDS_EN%20.pdf**
**Miscellaneous**	**[119-121],**<br><br>**[122], [123], [124], [125], [126], [127], [128]**<br><br>**Can J Psychiatry. 2012 Sep;57(9):537-46.**<br><br>**Epidemiology of postpartum depressive symptoms among Canadian women: regional and national results from a cross-sectional survey, $$$$ data from**<br><br>**Lancet. 2014 Dec 13;384(9960):2107-8. doi: 10.1016/S0140-6736(14)62367-9.Prevalence of overweight and obesity in children and adults. Keating C[1], Backholer K[2], Peeters A[2].**<br><br>**Global, regional, and national prevalence of overweight and obesity in children and adults during 1980–2013: a systematic analysis for the Global Burden of Disease Study 2013**<br><br>**[129],**<br><br>**Early childhood league service table from Innocenti UNICEF report 8, 10 benchmarks of advanced economies (early preschool enrolments taken from this report)**<br><br>***Innocenti Report Card 2***<br><br>**A league table of child deaths by injury in rich nations,**<br><br>***Innocenti Report Card 5***<br><br>**A league table of child maltreatment deaths in rich nations**<br><br>***Innocenti Report Card 7***<br><br>**Child poverty in perspective: An overview of child**

	**well-being in rich countries**<br><br>**Statens Serum Institut. Børnedatabasen [Children's database]. http://www.ssi.dk/Sundhedsdataogit/Registre%20og%20kliniske%20databaser/De%20nationale%20sundhedsregistre/Graviditet%20fodsler%20born/Bornedatabasen.aspx.**<br><br>**Autism Res. 2012 Jun; 5(3): 160–179. Global Prevalence of Autism and Other Pervasive Developmental Disorders**<br><br>**Mayada Elsabbagh, Gauri Divan, Yun-Joo Koh, Young Shin Kim, Shuaib Kauchali, Carlos Marcín, Cecilia Montiel-Nava, Vikram Patel, Cristiane S Paula, Chongying Wang, Mohammad Taghi Yasamy, and Eric Fombonne**

Table 3. CIMO-logic – the Components of Design Propositions (Denyer et al. 2008)

Component	Explanation
Context (C)	The surrounding (external and internal environment) factors and the nature of the human actors that influence behavioural change. They include features such as age, experience, competency, organizational politics and power, the nature of the technical system, organizational stability, uncertainty and system interdependencies. Interventions are always embedded in a social system and, as noted by Pawson and Tilley (1997), will be affected by at least four contextual layers: the individual, the interpersonal relationships, institutional setting and the wider infrastructural system.
Interventions (I)	The interventions managers have at their disposal to influence behaviour. For example, leadership style, planning and control systems, training, performance management. It is important to note that it is necessary to examine not just the nature of the intervention but also how it is implemented. Furthermore, interventions carry with them hypotheses, which may or may not be shared. For example, 'financial incentives will lead to higher worker motivation'.
Mechanisms (M)	The mechanism that in a certain context is triggered by the intervention. For instance, empowerment offers employees the means to contribute to some activity beyond their normal tasks or outside their normal sphere of interest, which then prompts participation and responsibility, offering the potential of long-term benefits to them and/or to their organization.
Outcome (O)	The outcome of the intervention in its various aspects, such as performance improvement, cost reduction or low error rates.

Table 4. Evidence for Effectiveness of Well Child Care Interventions**

WCC Intervention	Target population	Type of Research	Level of Evidence	Quality of Evidence	Outcomes/Size effects
**Open access scheduling**	Predominantly Mexican	RCT	II	Medium	Missed appointments reduced 21% to 9% , immunisation improved from 59% to 74%
**Primary care interventions for ECD***	0 to 3 years in 12 countries	Systematic Review *General development nine interventions(20* quasi experimental studies except Videotaping and co-interviewing parent child interactions (RCT) , parenting intervention (RCTs) *General behavioural 8 interventions(19 studies),* all at least one RCT *Specific developmental topic7 interventions(9 studies),* RCT only for colic interventions	II/III	Medium	2 interventions resulted in reduction in developmental delay, 4 improved cognitive scores, 6 reduction in behavioural problems (variable results, different outcome measures, Meta-analysis not possible)
**Developmental screening/counselling/referral at preschools**	Students enrolled in three school districts of Ontario	RCT	II	Medium	No difference in mental , social, behavioural well-being but increased worry among families/parents
**Improving continuity of care with same provider**	30 paediatricians at 11 practices	Cohort	III	Medium	Reduce ED visits ($r_{s=}$-0.242)
**Child health surveillance in UK****	59 schools reported children with PDD, and effectiveness of 2 and 3.5 years checks were	Retrospective cohort study	III	Low	94% children were identified at 3.5 yrs. check, and 13 (68.4%) were referred and therefore identified

	evaluated				
**Screening for developmental delays in paediatric clinics using screening questionnaires ASQs ****	Children's care clinic in Pierrefonds, Quebec Canada with seven full time paediatricians	Prospective cohort study, randomised	III	Low/Medium	Parent completed screening tools is feasible, but not enough psychometric properties
**Healthy Steps for Young children Program**	Perceptions of staff	Qualitative descriptive study	IV	Low	Staff positive about greatest benefit for low income families
**Personal Health Record use in Norway**	309 children attending Norway preschool surveillance program	RCT	II	Low/Medium	Acceptable to families and professionals but not influence the utilization of healthcare services, parents' knowledge of their child's health, or parents' satisfaction with information or communication with professionals
**Practice based education (audit, identify, and implement new processes) to improve anticipatory guidance**	44 paediatric practices in Carolina	RCT	II/III	Medium	At 6 month all age appropriate anticipatory guidance,2.2% (95% CI 0.8-5.9) to 18% (95% CI 10.3-29.9), no change in parent knowledge
**Primary care services for optimising child development**	literature review	2 RCTS- improving mother infant interaction<br>2 RCT- sleep problems<br>Group Well Child Care-2 RCTs<br>Book sharing 4 RCTs<br>RCTs- excessive crying, sleep and settling problems<br>Discipline-1 RCT<br>Television viewing -1RCT<br>Injury prevention-1 RCT targeted guidance	II/III	Low to medium	No concrete evidence for book reading, some evidence for promoting mother infant interactions, crying, sleep and settling problems , injury prevention,
**Systematic review for intervention**	17 RCTs		I		overall screen time reduction [-17.12 (95% CI -28.8 to -5.40], and sedentary time -18.91

**to reduce screen time and sedentary behaviours (0-5 yrs.)**	(n=22 to 885)				(95% CI -33.3 to -4.50
**Primary care interventions for reducing obesity (2-18 years)**	12 studies (10 RCTs)				small reduction in BMI z-score [-0.04, (95% CI -0.08 to -0.01)
**Well Child Care Clinical practice redesign**	Systematic review of strategies and tools	33 studies, 14 Healthy Steps, 8 group WCC, 5 non-face to face formats(2 RCTS, 2 observational studies, one controlled trial), 4 additional providers(One RCT), 1 home WCC, 1 preschool WCC-one RCT	II	Medium	Non face-to-face formats could enhance anticipatory guidance, involvement of non-medical but trained with knowledge of child development may improve receipt
**Educational kiosk in waiting rooms****	Controlled trial	52 parents Indigenous American Indian in control group along with 49 in intervention group	III	Low	Car seat use , dental care, nutrition, superior knowledge of parents
**Financial payment for families****	Low income families Financial payment with aim to encourage child health and well-being	Seven RCTs	I	High	No clear benefit that financial payments to low income families improve child health and well-being
**Identifying psychosocial determinants of health during well child care visits**	Well Child Care, Evaluation, Community Resources, Advocacy, Referral, Education [WE CARE])	RCT	II	Medium	WE CARE mothers received ≥1 referral at the index visit (70% vs 8%; adjusted odds ratio [aOR] = 29.6; 95% confidence interval [CI], 14.7–59.6). At the 12-month visit, more WE CARE mothers had enrolled in a new community resource (39% vs 24%; aOR = 2.1; 95% CI, 1.2–3.7
**Multi-sectoral group discussion and interactive lecture based program ****	Salut programme in Sweden in 13 municipalities	Qualitative evaluation	III	Medium	An increase in the extent to which (1) midwives in antenatal care raised issues related to men's violence against women, (2) an increase in the extent to which several

					lifestyle topics were raised with parents/clients in child health care and dental services, (3) an increased use of motivational interviewing (MI) and separate 'fathers visits' in child health care (4) improvements in the supply of healthy snacks and beverages in open pre-schools and (5) increased collaboration between sectors
**Perry Preschool Program****<br><br>**Hawaii's Healthy Start****<br><br>**Dublin Community Mothers' group****<br><br>**Home Nurse visiting programs::**<br><br>**Parent education programs(Positive parenting program)****	Mostly vulnerable populations in multiple countries	Literature review on risk factors, interventions for early childhood development	III/IV	Low to Medium	Preschool enrolment particularly for low income families improve short term cognitive gains<br><br>Specific targeted programs improve immunisations rates, appropriate feeding, improvement in parenting skills, reduce child abuse and neglect in low income families , improvements in children's behaviour<br><br>Cost effectiveness in terms of later returns in invested money
**Survey Dutch preventive child health system****	2229 children within Dutch child health surveillance program	Survey of parents and evaluation of Dutch program	III	Medium	9.4 % of all children psychosocial problems were identified
**Meta-analysis and systematic review [59]**	Parenting education for injury prevention	Nine RCTs	I/II	Medium	Mean risk reduction 18 %(95% CI 5- 29%)
**Literature review ****	Young, first time mothers	Family nurse partnership program- three RCTs<br><br>Tripe P<br><br>Incredible years parent training	II	Medium	48% reduction in child abuse and neglect, 56% reduction in emergency room visits, 67% reduction in behavioural problems<br><br>Child maltreatment in intervention group (11.7 vs 15.06 per 1000 children)
**Meta-analysis**	Any parent with concerns about their child's behaviour	50 intervention studies with control group	III	Low to Medium	mean effect size of d = .27 concerning disruptive child behaviour across informants
**Meta-analysis**	Children with parenting, behaviour problems	55 intervention controlled trials	II	Medium	Parenting (overall ES = 0.38), Child Problems (overall ES = 0.35), and Parental Well-Being (overall ES = 0.17)
**Evaluation of program****	Christchurch Sure start	Home visitation program	III	Low to Medium	More visits to GPs, reduction in hospital

	Home based family support programme, home visitation program				attendance (26.3% vs 17.5%), attendance at early childhood education (90.8 Vs 84)
**Systematic review**	Education, counselling, documentation, behaviour change strategies for obesity in preschool children	RCTs	II	Medium	Short term changes in weight
**Cohort study****	Implementation of safety measures by Swedish municipalities	All 25 Swedish municipalities	III	Low to medium	Risk of injury was 33% higher in municipalities which did not implemented safety measures

*ECD-Early Childhood Development, PDD-pervasive developmental disorders, **Details of studies are listed in Appendix Table 5 with a complete reference list

Table 5 Reviewed literature on Well Child Care using realist approach of Context, Mechanisms and Outcome

Study type	Reference	Country of origin	WCC intervention	Local context<br>What?	Whom?<br>Mechanism	Outcome
**Cluster randomized controlled trial**	**[1]**	**USA**	**Effect of Open access scheduling on missed appointment/immunizations and continuity of care**	**Community health centre within an integrated health system in Denver**	**10 Paediatricians and**<br>**Physician assistants/open access system**<br>**Predominantly Mexican population**	**Missed appointments reduced 21% to 9% , immunization improved from 59% to 74%**
**Evaluation study**	**[2]**	**USA**	**Participation in Breastfeeding practices/Well child care for women in Special Supplemental Nutrition Program for Women, Infants, and Children (WIC)**	**Part of larger Fragile Families and Child Well Being Study**	**Unmarried Low income mothers residing in 20 cities of USA**	**Increased well child visits but not breastfeeding initiation**
**Evaluation study**	**[3]**	**USA**	**Impact of parent education on child development outcomes at 30 months**	**80% of fee for service paediatricians in city and suburbs of Rocehster, New**	**Mothers of first born children in Rochester New York**	**No positive relationship between physician education and child**

				York		development, more behaviour problems in teaching group
Evaluation study	[4]	USA	Effect of insurance on access, utilisation and satisfaction of WCC	Analysis of nationally representative of Medical Expenditure Panel survey	12, 270 children (2-17yrs) across USA	Children with gaps in insurance coverage less likely to have usual provider and WCC
Randomized controlled trial	[5]	Canada	Developmental screening/counselling/referral of preschool children	Public health school registration clinics in three of four school districts in Niagara region (Southern Ontario)	4797 4-5 yrs registering	Not effective in improving school performance, behavioural or developmental outcomes in early school years
Census of child development	[6]	Australia	Developmental vulnerabilities	Teachers completed Australian Early Development Index for children	261 147 children in first year of school (97.5% 5 years)	Demographic and socio economic inequalities within and between jurisdictions
Evaluation study	[7]	USA	Infant continuity of well child care and emergency (ED) visits	Children enrolled by Children's Medical Group (11 clinic sites and 30 paediatricians) in greater Milwaukee metropolitan area	181 children less than 18 months	Improved Continuity of WCC reduced ED visits
Evaluation study	[8]	UK	Developmental surveillance in general practice	2157 children enrolled in a single general practice (six man) in South England	1775 children <5 yrs five routine surveillance at 7 months,12 mo, 28030 mo, 3 yrs and 41/2 yrs	Suspected disorders in 15% boys and 11% girls
Implementation study	[9]	USA	New internet based model for delivery of WCC	2 practices within Kaiser Permanente Colorodo serving 100000 children in Denver	70 parents and seven providers	In qualitative evaluation 12% parents found the online assessment somewhat or very difficult, most found evisit acceptable
Survey of parents	[10]	USA	Parenting knowledge of child development	Evaluation study of three main immigrant groups in Washington metropolitan area	114 mothers of 20 month old infants from Japanese, South America and European Americans	Immigrant mothers scored lower than multigenerational US mothers and mostly answers were incorrect on normal child development
Evaluation study	[11]	USA	Rates of parent-centred development screening	Evaluation of parent completed standardized	22 883 children less than 71 months	Only 19% were screened and the figure varies widely

				questionnaire using Nationally representative sample of US children (2007 National Survey of Children's health)		10.7-47% between states
Evaluation study	[12]	USA	Physician practice patterns and variation in the delivery of preventive services	Analysis of data of 4454 outpatient visits to family physicians in Ohio from Direct Observation of Primary Care (DOPC)	Six practice configurations were identified based on based predisposing, enabling and reinforcing characteristics for preventive care	No differences in well child care according to practice configurations
Survey Evaluation	[13]	USA	Evaluating provider perspectives on WCC	31 focus groups of 282 paediatricians and 41 nurse practitioners in 13 US cities	Providers of WCC physician led 86%, in managed care 60%, group practice 39%, urban 58%, rural 10%	Suggest Innovations in practice organization, community linkage, education and enhanced resident training in developmental behavioural paediatrics
Epidemiological study	[14]	UK	Evaluate role of 2 years and 3.5 years health surveillance checks on early detection of children with pervasive developmental disorders (PDD)	Year 4 School children in the Maidstone district of Kent with a diagnosis of PDD	Preschool children managed with routine child health surveillance	13 children out of 33 children were referred as a result of preschool check and were identified early
Questionnaire survey	[15]	Canada	Evaluate risk factors for developmental problems at 3 years of age	791 Low risk mothers from Calvary region of Canada recruited in a randomized trial were followed up for child development at 3 yaers	Mothers identified with mental health problems	10% of children at risk and mental/emotional health increased risk
Literature review and opinion piece	[16]	USA	Extensive review of literature, 179 articles reviewed	Summary of Evidence/Well child care scenarios	Predominantly focused on USA	Preventive Services provided not consistent,<br><br>need to address specific population needs,<br><br>Identify psychosocial problems more, address maternal health needs, use screening tools to identify developmental behavioural problems,<br><br>Home visitation for

						targeted groups<br><br>Use innovative strategies to improve delivery
Opinion piece	[17]	Australia	Perspectives on the recent proposed changes in the 4 year old healthy kids check	National federally funded item for primary General practitioners	To screen 3.5 to 5 years children for socio-emotional, developmental, vision, hearing and other problems and other well child care	Uncertainty, implementation trial completed in some regions
Australia<br><br>Mental health provisions in the 2011-2012 budget	[18]	Australia	Commentary on allocation of budget for mental health by allied health and GPs	Rational of budget allocation for 4 year healthy kids check	Uptake of 4 year healthy kids check, linked with family tax benefit Part A (welfare payment) to enhance uptake	In 2 year period July 2008-June2010 only 81,463 healthy kids check done against expected 2,60,000, most done by practice nurses
Theoretical model	[19]	USA	Systems model of clinical preventive care	Parent –physician interaction crux<br><br>Both influenced by predisposing factors (beliefs), enabling factors (abilities), and reinforcing factors (rewards)<br><br>Independently influenced organizational factors, preventive factors and situational factors		Preventive behaviour of users and providers
Evaluation study	[20]	Canada	Responsive Interdisciplinary Child-Coordinated Community Health Education and Research (RICHER)- an inter-sectoral and interdisciplinary community outreach primary health care model with embedded Social Paediatrics (SPI) clinic	One of Canada's poorest area adjacent to Vancouver downtown eastside	86 patients were surveyed and 7 were interviewed	Mean clinic use 7.2 times/12 month<br><br>61.6% access same day<br><br>54.7% development assessed<br><br>19.8% referred<br><br>81.4% same provider always or usually, compassionate interpersonal style

**Prospective evaluation of screening tool**	**[21]**	**Canada**	**Screening for developmental delays in paediatric clinic using parent questionnaires**	**Children's care clinic in Pierrefonds, Quebec Canada with seven full time paediatricians**	**317 parents returned Ages and Stages questionnaire or child development inventory**	**Parent completed questionnaires are feasible, but screening tools don't meet psychometric properties**
**Descriptive study**	**[22]**	**USA**	**Adolescent mothers perceptions about their children development**	**Adolescent mothers attending high school with children attending child centre within the school in New England USA**	**45 adolescent mothers with <2 years old children**	**73% accurately assessed on ASQ and 18% identified delays when none existed**
**Opinion group**<br><br>**Canadian taskforce on Preventive Health Care**	**[23]**	**Canada**	**Taskforce of 7 family physicians, and remainder other medical specialists, allied health practitioners with interest in preventive care and methodology**	**Systematic evidence for guideline development taking methodology, grading evidence, considering contextual factors, knowledge transfer and performance measurement**		**Guidelines for preventive care for children in Canada**
**Evaluation study**	**[24]**	**USA**	**Healthy Steps for Young Children Program**	**Assessing changes in provider perceptions for developmental services for low income children**	**56 Providers from 20 sites participating in a national Healthy Steps program**<br><br>**13 sites in urban areas,5 small cities and 2 rural areas at two time points**	**Across all income groups more preventive development services were provided, major barriers for low income families reimbursement and time**
**Evaluation study**	**[25]**	**USA**	**Evaluating barriers of well child care in homeless children**	**Homeless families three transitional homeless shelters in two neighbouring southern Californian counties**	**53 families with 120 children, 51 children <5 years**	**Provider selection difficulties, waiting for and then during appointments and transportation/parking costs**
**Guidelines**	**[26]**	**Canada**	**Update on nationally endorsed Rourke Baby record for WCC**	**Growth monitoring using WHO charts instead of CDC, injury prevention aspects enhanced, safe sleeping room sharing protective, encouraging parents to read, aspects of high quality child care, pacifier use not be discouraged, fluoridated toothpaste, vision and hearing screen, Ontario version include Nippising**	**Both parents and providers**	**unknown**

				screen		
Position Statement	[27]	Canada	Including 18 months enhanced visit particularly in Ontario using a validated Nippissing District Developmental screen	Ontario<br><br>Web based portal developed for parents<br><br>Nippising screen provided free to residents of Ontario	18 months<br><br>Physician generated and provided fee code	18 month population health measures under development
Randomized controlled trial	[28]	USA	An office based intervention to improve delivery of anticipatory guidance	49 paediatric practices in North Carolina<br><br>Improvement team of clerical, nursing and physician staff members	Intervention included prevention coordinator, prompting system, preventive service summary , a tracking recall system for 357 parents of 1 and 6 months attending WCC	Anticipatory guidance improved from 7.3% to 24% and from 2.2% to 18.1% for parents of 1 months and 6 months respectively
Evaluation study	[29]	USA	Evaluating racial difference in compliance of WCC guidelines	National Maternal and Infant Health Survey 1988 representative sample of all live births in US	Maternal and provider reports on compliance of WCC visits	African Americans, low maternal education, poverty, inadequate prenatal care, maternal depression, unwanted pregnancy, b wt. less than 1500 grams were risk factors
Opinion piece	[30]	UK	Need and role of a coordinator in child health surveillance/promotion	Suggestions for child health surveillance for UK, not prescriptive for local contexts	Outlines the expected role of a coordinator for child health surveillance	Not applicable
Evaluation study	[31]	Australia	Developmental behavioural problems in paediatric office settings using paediatrician reports of 100 consecutive consultations	Paediatrician provisions for developmental problems	196 paediatricians (66%) of the 300 eligible members of Australian Paediatric Research Network	Developmental problems accounted for 1/3rd of consultations and required average 9 mins longer, parents reported worse health , referrals were common, Baby check required for 5% of medical consultations
Evaluation study	[32]	USA	Adherence to AAP guidelines under managed care	Insurance claims data evaluated for 130 572 children living in Monroe county, New York	Children enrolled in private health insurance and Medicaid covering more than 70% of population	46% of private insurance and 35% public insured received recommended visits
Evaluation study	[33]	USA	Evaluating nurse practitioner-	Nursing clinic in a largely	Majority of visits for children less than 10	Nursing practitioners

			patient interactions	Latino community in Texas<br><br>Half walk ins and half scheduled appointments	years	delivered primary care in a friendly style
Evaluation survey	[34]	Australia	Findings from NSW child health survey 2009-2010 for regularly seeing baby or childhood nurse	Access to early childhood nurses	Access by mothers for children 0-11 months and 1-4 years according to socioeconomic disadvantage	60% mothers regularly see early childhood nurses up to 11 months and this reduces to 18.4% between 1-4 years
Review of literature	[35]	USA	Literature reviewed up to 1999 for office settings for effectiveness of education, intervention and care coordination	Not applicable	Systems, users and providers	Assessment of parental concerns and psychosocial risk factors more accurate in identifying developmental problems than clinician appraisals, primary care activities for crying, sleep problems, parent child interactions are effective
Evaluation study	[36]	Australia	Parents and carers of children evaluated for utility of Parents Evaluation of Development Status (PEDS)	262 children attending five day care centres and two kindergarten between 18 months and 5years and 9 months in Melbourne	Parents and carers of young children	98% liked the PEDS and 9% classified at high risk, while 19% medium risk
Systematic review	[37]	Australia	Systematic review of literature to understand the parental concerns of development	37 studies with total of<br><br>210,242 subjects reported concern for their child	parents	13.8% children indicated high risk and 19.8% indicated moderate risk for their child development
Prospective randomized study	[38]	USA	Evaluation of patient specific letters describing the content or postcard reminders of upcoming WCC visit on show rates	Paediatric continuity clinic in Rochester, New York<br><br>Care provided by paediatric residents, population served include 7300 predominantly poor urban , 71% visits by Medicaid recipients	288 children for well child care	Show rate improved between 73.7 to 75% for intervention groups
Evaluation study	[39]	Australia	Comparison of health status and service utilisation of indigenous	5107 families enrolled in first wave of Longitudinal	154 indigenous infants were compared with 3746 non-indigenous infants	Indigenous infants less likely to use maternal and

			**and non-indigenous infants**	**Study for Australian Children (LSAC)**		**child health centres or nurses or helplines, GPs or paediatricians, Financial status and location were predictors for service use**
**Evaluation study**	**[40]**	**Australia**	**Health service utilisation disparities between non-English and English speaking Australian infants**	**Families enrolled in LSAC**	**374 infants from non- English versus 3700 English speaking infants, also data analysed according to mothers limited English proficiency**	**Non English speaking infants less likely to use maternal and child health centres or nurses or helplines, GPs or paediatricians, SE status and language barrier only partial explanation**
**Review and opinion piece**	**[41]**	**USA**	**Evidence for anticipatory guidance reviewed up to 2003**	**Missed opportunities for anticipatory guidance**		**Anticipatory guidance improve function outcomes of children and their families, injury prevention and reading at home is supported by evidence**
**Opinion Piece**	**[42]**	**Australia**	**Opinion leaders views on preventive health reform in Australia**	**National and states**		**Success requires whole of government approach and long term investments, a sizeable proportion outside Health in social, economic and environment, how to best integrate and outcomes**
**Systematic review**	**[43]**	**USA**	**Systematic review of strategies and tools for clinical practice redesign for well child care**	**Evidence**<br><br>**33 articles selected , 17 provider, 13 formats, 2 location and 1 miscellaneous**	**Systems , providers and users**	**WCC in groups effective, non-face to face formats, web based tools enhance anticipatory guidance, addition of a non-medical professional trained enhance parenting practices**
**Implementation and evaluation study**	**[44]**	**USA**	**Implementation and impact of a novel method using educational kiosk (computerized tutorials)**	**Public funded facility for Navajo patients , part of US Indian health service for natives in New Mexico**	**101 Navajo parents with young children**	**Superior knowledge of parents in WCC components such as car seat use, dental care, nutrition, fever under 3 months**
**Evaluation survey study**	**[45]**	**USA**	**Paediatrician reported practices regarding developmental**	**National survey of paediatricians in USA**	**894 paediatricians, 646 practising general paediatrics, 21% urban inner city, 27%**	**71% used clinical assessment and only 23%**

			screening		urban, non-inner city, 40% suburban and 12% rural	used screening instrument
Evaluation study	[46]	USA	Maternal and provider characteristics for completion of immunization at 19-35 months of age	Data from 2003 National Immunization Survey	9510 children not up-to-date at 18 months and 2350 up-to-date	Children with less educated mothers and Hispanic and Non-Hispanic Black families with low income more up-to-date, likely due to access of government subsidized programs
Review of literature/opinion piece	[47]	Australia	Review of literature to understand nature and impact of collaboration and integrated service delivery for pregnant women, children and families	Evidence	Systems, providers and users	Collaboration of universal health services move from co-existence to models of cooperation and coordination
Evaluation of national program	[48, 49]	UK	Evaluation of national level Sure Start Local programmes (SSLP) of integrated early education, child care, healthcare and family support services in disadvantaged areas of UK	5000 families in 150 SSLP areas with children at 7 years (initially studied 9 months, 3 and 5 years)	Families with young children from disadvantaged areas	Mothers engaged in less harsh discipline, more stimulating environments for children, less chaotic home environments for boys and better life satisfaction
Evaluation of state program	[50]	Australia	Evaluation of state-wide (Victoria) program of Best start program for vulnerable and at risk families	State program funded by government<br><br>Best start facilitators<br><br>Service co-operation and co-ordination tool<br><br>Survey of parents	11 disadvantaged areas based on Index of Relative Social-economic Disadvantage (IRSED) and 2 indigenous sites, 6 regional/rural and 6 metropolitan areas	Improved service cooperation and partnerships, improving maternal and child health attendance, ongoing indicators

**Descriptive paper**	**[51]**	**Australia**	**Discusses 10 integrated models**	**Sure Start (UK)**<br><br>**Every Child matters (UK)**<br><br>**First Duty Toronto and Healthy Child Manitoba (Canada)**<br><br>**Head start & Early Head start (USA)**<br><br>**First 5 California (USA)**<br><br>**Stronger families and communities (Australia)**<br><br>**Best start (Victoria)**<br><br>**Families NSW (NSW)**<br><br>**Every Chance for Every Child (South Australia)**	**Sustained integration of all program "top down" and "Bottom up" activities**<br><br>**Changes at planning, policy and practice levels**<br><br>**Negotiation of differences between agencies (values, power and priorities)**<br><br>**Co-location**<br><br>**Multiagency teams, clear protocols**<br><br>**Joint training**<br><br>**Information sharing**	**Only high quality service improve some outcomes**<br><br>**Mixed results on child outcomes (often difficult to follow longitudinal outcomes/lack of randomized data), perhaps narrowing of population outcomes at middle childhood**<br><br>**Expanded roles of nurses, social workers, teachers**<br><br>**Challenges of integrating teams**
**Literature review**	**[52]**	**Australia**	**Evidence regarding use of Child Health Records (CHR)**<br><br>**Purposes of CHR children, parents, health system**<br><br>**Existing CHR models**	**Evidence**	**Systems, providers and users of WCC**	**No evidence of CHRs in improving health outcomes**<br><br>**May be associated with more up-to-date immunisation status**

						**Useful tool for parent education, engagement and communication**
**Opinion piece**	**[53]**	**UK**	**Authors views on the changes in UK child health surveillance (CHS) program of proactive screening process to passive child health promotion (CHP)**	**Changes in the UK system of delivery of Well child care**	**Systems**	**Reliance on cares may potentiate "inverse care law" and exclude children who need most input**
**Cluster randomized controlled trial**	**[54]**	**USA**	**127 practices within the Paediatric Research In office settings (PROS) of AAP intervention included an office based violence prevention intervention**	**Clusters of paediatric office practices based on rural versus urban and service to ≥ 50% minority patients**	**Primary caregivers of children aged 2 to 11 years**	**Significant effects at six months for decreased media use and safe firearm storage**
**Evaluation study**	**[55]**	**USA**	**26 practices from PROS were recruited and parents and providers were asked if age appropriate anticipatory guidance topics were discussed**	**Evaluation project for number of anticipatory topics**	**32 practitioners and 861 parents of children aged 2 to 11 years**	**Providers discussed nutrition, car restraints, dental care, and reading aloud, high concordance between providers and parents, but parental recall decreased if ≥9 topics are discussed**
**Evaluation study**	**[56]**	**USA**	**Development of promoting development survey to evaluate health care system performance**	**Evaluation of system for providing family centred anticipatory guidance and child and family assessment services for children up to 48 months**<br><br>**3 managed care health plans**	**System and providers and users**<br><br>**580 parents**	**Seven quality measures for system performance were developed for anticipatory guidance**
**Evaluation study using survey**	**[57]**	**USA**	**Evaluation of information received by parents as compared to what they want**	**Commonwealth fund developed survey**	**2017 parents of young children who have received WCC**	**Majority of parents reported that they had not discussed important age related anticipatory guidance topics**
**Implementation study**	**[58]**	**USA**	**Implementing and studying impact of structured encounter form on provider performance for WCC**	**Harriet Lane primary care program of John Hopkins hospital, University based group practice**	**1031 visits by parents to 68 house staff providers**<br><br>**Direct observation of 243 encounters**	**Structured form improved recorded and observed performance**

**Qualitative Evaluation study**	**[59]**	**Sweden**	**Primary health care nurses assessment of risks in children of foreign origin**	**Primary health care nurses perceptions of working of immigrant families and children in a Swedish county**	**Fifteen Primary Child health care nurses**	**Felt anxious about missing issues in children exposed to risks in children's home environment**<br><br>**Difficulties in making psychosocial stressors due to unfamiliar to assess**
**Implementation/**<br><br>**Evaluation program**	**[60]**	**USA**	**Evaluate WCC delivered by paediatric nurse practitioners (NP)**	**474 by the NP and 678 by paediatricians at Kaiser Permanente Medical centres at San Francisco and Oakland**	**1152 children up to 22 months receiving well child care**	**NP were fully competent and acceptable to parents for WCC**
**Evaluation study**	**[61]**	**UK**	**Factors affecting GPs uptake of Child health surveillance (CHS)**	**Three diverse districts England in terms of socioeconomic indicators (SES)**	**399 GPs out of which 108 GPs provided CHS (62 female & 46 males)**	**More GPs in higher SES districts, more females and GPs with training in paediatrics provided CHS**
**Evaluation study**	**[62]**	**Australia**	**Nature and frequency of services by child and family nurses (CHoRUS study)**	**Roles delineation for well child care**	**1098 child and family nurses Australia wide**	**82.8% reported first contact with families within 2 weeks (80.7%) in Services mostly at health centre but 25% reported other locations (parks, preschools).**
**Evaluation study**	**[63]**	**USA**	**Utilisation of WCC services for late preterm and full terms receiving Medicaid (low income families)**	**South Carolina population based study**	**25,940 children in South Carolina, out of these 1956 late preterm (LP)**	**LP children use more healthcare services in first 6 yrs, and those making recommended WCC visits ED less for non-ambulatory conditions**
**Early childhood literature review up to 2000**	**[64]**	**Australia**	**Risk and protective factors studies**<br><br>**Kauai Longitudinal study (USA)**<br><br>**Mater 900 Queensland**<br><br>**National Child Development study (UK)**	**Evidence**	**Early childhood**	**Enumerates various risk and protective factors**<br><br>**Attendance at preschool/quality childhood program improve outcomes more so for disadvantaged populations**

			**Dunedin Multidisciplinary Health and Development Study , New Zealand**<br><br>**California child health and development study USA**<br><br>**Christchurch Child Development study**<br><br>**Australian Temperament Project**<br><br>**Intervention studies**<br><br>**US review 15 studies in 13 countries on early childhood systems [65]**<br><br>**US review of 15 small scale programs/21 large scale programs centre based education, home visiting, parent support, preschool[66]**<br><br>**Child health surveillance studies**<br><br>**Review of studies on anticipatory guidance[67]**<br><br>**Home visiting program studies**<br><br>**Hawai Heathy start**<br><br>**Teachers as parents**<br><br>**Comprehensive Child development study**<br><br>**Dublin community Mothers program**<br><br>**Home visitation program**			**Anticipatory guidance promotes child development**<br><br>**Home visiting (HV)programs are useful for select groups and increase IQ during early years, improve reading and maths achievement, grade retention and socialisation, parenting skills, reduce child abuse and neglect**<br><br>**conflicting unsuccessful HV programs are also highlighted**

			Home Instruction program for Preschool Youngsters			
Evaluation study	[68]	USA	Parenting sources of information	Cross sectional analysis of mother-infant dyads enrolled in a larger longitudinal study FROM Bellevue hospital, urban public hospital serving at risk	Low SES mothers	
Evaluation study	[69]	Denmark	Early predictors of psychopathology in children at 18 months	Child health surveillance reports by Danish nurses at 0-6 months, 8-10 months with mental health problems	210 children of 18 months in Copenhagen Child Cohort (CCC2000) identified with mental problems	Deviant language at 10 months predicted problems at 18 months, impaired social interaction and communication in first 10 months predicted neurodevelopmental disorder at 18 mos
Evaluation study	[70]	Netherlands	Identification And management of psychosocial problems in toddlers in Dutch preventive system	16 of 65 child health centres selected by stratified sampling	2229 children aged 21 to 48 months	9.4% children with problems were identified, many children with parent reported problems were missed
Evaluation study (survey)	[71]	USA	Understanding physicians practices for identification of developmental delays	National survey of 341 paediatricians and 199 family physicians	Provider practices/perceptions	Reviewed developmental milestones, half used screening tool, females used screening tools more
Opinion Piece	[72]	USA	Issues of WCC in USA	Academic practice	Opinion leader views	Call for major revision of WCC, variability of delivery, less used components WCC
Evaluation study	[73]	USA	Secondary analysis of Heathly Steps for Young Children	Practice based intervention to promote parental practices	4753 families at 24 paediatric practices across US with young children <3 yrs	96 % parents satisfied with care, parents reporting negative experience, led to reduced utilization and less chance of up-to-date with 2 year immunization
Descriptive study	[74]	Multi-country	Description of variability of	Paediatric primary care in	Systems and providers	Variation of roles for WCC

		authorship	paediatric primary care in Europe	Europe (29 countries)		within European union nations
Evaluation study	[75]	Denmark	Factors for non-participation in a Dutch preventive system	Danish registry analysis of socio-demographic factors for non-participation in preventive system	Users factors	Young , single, un-employed parents, with low education and less income with more children accessed the services less
Evaluation study	[76]	Australia	analysis of nationally representative data on length/patterns of GP consultations in Australia	National GP consultations with children	Systems and providers	Despite increase in children populations, GP longer consultations have reduced and become shorter in duration
Literature review Meta-analysis	[77]	Australia	Effectiveness of Tripe P parenting programs on parental and child well being	Evidence	Systems and providers	Moderate effect on outcomes on parent and child well-being, long term data after three years lacking
Survey Evaluation study	[78]	Sweden	Providers perspectives on WCC components of Swedish guidelines	Evaluation of 34 chief medical officers representing 1731 CHC in Sweden	Providers , systems	National guidelines for WCC followed closely, breast feeding rates satisfactory at 4 months
Literature review	[79]	Switzerland	Screening programs evidence for speech, language, behaviour, motor, anticipatory guidance	Systematic review of evidence	Influence on academicians and policy makers	Uptake of screening drops after 2 years, anticipatory guidance improve parental educational competencies, early motor/cognitive developments later development
Literature review	[80]	UK	Effectiveness of parenting interventions to promote child health<br><br>*Family Nurse partnership(FNP)*<br><br>*Triple P positive parenting program*<br><br>*Incredible years program (IYP)*	Three parenting interventions for vulnerable mothers in UK	Influence on academicians and policy makers and providers	FNP- 67% reduction in behavioural and intellectual problems, 56% less ED visits, 72% fewer conviction of mothers, 48% reduction in child abuse and neglect<br><br>*Other programs-* improved parenting, well-being, and stress
Review	[81]	UK	Health services systems for	Comparison of health	Policy makers and providers of service	Whole-systems approach

**article/opinion piece**			**children in Western Europe**	**systems for reconfiguring child health systems to meet contemporary challenges**		**with three interdependent systems-practice (first contact, chronic care, training needs), plans (research, analysis, indicators), policy (goals, transparent structures, commitment)**
**Commentary/Opinion piece**	**[82]**	**UK**	**Overall immunisation, screening, health promotion and parental support**		**Policy makers and providers**	**Call for more local data, importance of getting to know local data**
**Literature review/opinion piece**	**[83]**	**UK**	**Review of evidence on obesity, developmental and behavioural problems and injury**	**Intention to guide the new CHP programme which was launched in 2009**	**Programme makers and providers**	**Benefits and harm of universal and targeted system discussed**
**Literature review**	**[84]**	**UK**	**Review child health promotion programs in select countries**	**Review to compare UK CHP program to other developed economies of Australia, Canada, USA and Sweden**	**Program makers particularly of UK**	**All countries provided CHP services child health reviews, immunizations, screening to pre-schoolers, variability due to contextual factors**
**Cost analysis of new WCC model**	**[85]**	**USA**	**New model based on internet technology, web based pre-visit, different types of visits, brief vs e-visits and extended visit for children with special care need with extended visits**	**14,910 paediatric enrolees, ages newborn to five years in Colorado, Denver**	**Providers and policy makers**	**Cost neutral way could be provided**
**Opinion piece**	**[86]**	**Sweden**	**Elaborates the reasons for success of early child development programming**	**Opinion leaders**	**Policy makers/politicians**	**Political framework of socialist approach**<br><br>**A positive view of state by Swedes**<br><br>**Individualist attitudes and women's and children's rights**<br><br>**Decentralization of services in 290 municipalities**
**Evaluation Report**	**[87]**	**New Zealand**	**Home based family support program**	**Evaluation of a home visitation "Early Start" Program in Christchurch**	**Vulnerable families , providers**	***Child related outcomes***<br><br>**Improved Use of WCC and**

						**dental checks , fewer attendances for accidents, injuries, poisonings**<br><br>***Maternal related outcomes***<br><br>**Little apparent benefit in maternal and family outcomes**
**Framework for Child health and Development**	**[88]**	**Australia**	**Five dimensions of Dimensions of child development and five stages for healthy development**	**Evidence/theoretical framework**	**Providers, program makers, academicians**	**Describes the 5 developmental dimensions which requires different social structures at five different stages of life**
**Marmot Review**	**[89]**	**UK**	**Describes Health inequalities**	**Evidence**	**Policy makers, program developers, users and providers**	**Highlights the inequalities between child development indices such as B weight, postnatal depression, read every positive parenting practices supporting child development, school readiness**
**Evaluation**	**[90]**	**Australia**	**Evaluates first time mothers experiences of a facilitated peer support groups (Early Bird Program), positive parenting, family support**	**First time Mothers enrolled in a supported peer program aimed at promoting parenting skills**<br><br>**Early Bird facilitator**	**40 first time mothers**<br><br>**Providers of nursing services/policy makers**	**Group approach helped to empower mothers and improve confidence**
**Descriptive Survey**	**[91]**	**European Union Network**	**Describes the demography of the primary care paediatricians using survey of in 41 European Paediatric societies**	**Role provision/training needs**	**Policy makers, academicians, universities/professional organizations**	**Primary care paediatric system 12 countries, 6 countries GP system, 16 countries combined**
**Systematic review**	**[92]**	**UK**	**Describes the factors explaining social inequalities in childhood development across Europe**	**Evidence**	**Policy makers, academicians**	**Neighbourhood deprivation, lower parental income/wealth, educational attainment, and occupational social class, higher parental job strain, parental unemployment, lack of housing tenure, and household material deprivation were identified**

						**as the key social factors associated with a adverse child health and developmental outcome**
**Review of literature**	**[93]**	**UK**	**Insights from European child health systems to improve child health in the UK**	**Evidence describing primary care child systems**	**Policy makers, providers**	***Sweden*- first access by GPs working with paediatricians (co-location) and local nurses, “chains of care” based arrangement**<br><br>***Netherlands*- general practice system, but trans-mural design**<br><br>***France*- incentives to register with GPs, young children seen by paeds and GPs**<br><br>***Germany*- primary care paediatrician, more incentives now for GP**
**Models of care for Description of Model**	**[94]**	**Australia**	**Describes the role of aboriginal models of care to improve child and mother health outcomes for aboriginal population in Western Australia**	**Evidence**	**Policy makers and program developers and users and providers**	**Outcome indictors described to be improved**
**Evaluation study**	**[95]**	**USA**	**Parenting skills and confidence in Group well child care visits**	**North Carolina based group WCC visits**<br><br>**Groups with 4-8 mothers with their infants, facilitators, physicians, midwife**	**Providers and users, young mothers**	**Mothers reported support from other women, learning from others experiences, more time and parental involvement**<br><br>**Less ED and ambulatory visits**
**Framework/ State policy document**	**[96]**	**Australia**	**Describes Maternal and Child Health Service Guidelines**	**Policy framework for Victoria maternal and child health service, new initiatives**	**Policy makers, providers and users**	**[participation rate in the service**<br><br>**Use of Personal Health**

						record for Out –of –Home care in children
Systematic review	[97]	USA	Review of literature to understand universal mental health screening in paediatric primary care	Evidence search	Policy maker, program makers, providers	Little known about how best to engage parents, who administers (and is he best suited) , how is the purpose and process of screening explained, who assist CALD groups families with completion, , how providers explain and confirm screening results with families
Opinion piece	[98]	UK	Describes development in child health surveillance in UK	Opinion	Policy makers and providers	Highlights the central role of Nurses in UK CHS system
Government document	[99]	South Australia	Explores family Nurse Home Visiting program for aboriginal and Torres strait islander people	Pilot study of views of parents of Aboriginal children and organisation factors	Providers and policy makers	Program outcomes to be developed
Evaluation study	[100]	Sweden	Explores municipality level safety measures effect on childhood injuries	Health promotion program for injury prevention	Policy makers, providers and public	Municipalities implementing few safety measures had 33% higher risk of injury
Evaluation study	[75]	Denmark	Explores factors associated for non-participation in Dutch preventive system	Non participation in Dutch preventive system	Policy makers and providers	Young single parents with less income, low education and unemployed less attended preventive child health
Position/Consensus group Statement	[101]	European Union authors	Representatives from 17 countries of European Economic Area discussed the Child health indictors	Evidence	Academicians, policy makers and providers	Several child health indictors from Demography, child health status and well-being, child morbidity, risk and protective factors, health system and policy indictors were outlined
Evaluation study	[102]	Australia	Evaluation of identification and management of children after introduction of 4 year screening	Two Queensland practices, retrospective review of case records, no comparator	Policy makers and providers	GPs are using appropriate clinical judgement during HKC , 3% children

			**Healthy Kids check (HKC)**			**management changed because of HKC**
**Descriptive of health system**	**[103]**	**France**	**Describes issues of Child health services in France**	**Mandatory exams at 8 days, 9 months and 2 years linked to child benefit allowance**	**Policy makers and providers**	**Not relevant**
**Opinion piece**	**[104]**	**UK**	**Describes the changes in the UK child health surveillance program**	**Evidence**	**Policy makers and providers**	**Not relevant**
**Evaluation study**	**[105, 106]**	**Sweden**	**The tools used for screening mono and multi-lingual Swedish children**		**Providers and program makers**	**Multilingual 2.5 and 3 year old Swedish are not screened with same quality as monolingual Swedish speaking peers**
**Evaluation study**	**[107]**	**Netherlands**	**Evaluation of the Dutch child health surveillaince system**	**Review of system**	**Providers and policy makers**	**Demonstrated need for cost-effectiveness ratios**
**Evaluation study**	**[108, 109]**	**Australia**	**Health professional perceptions for continuity of care**	**132 professionals including 45 midwives, 60 child and family nurses, 15 GPs and 12 practice nurses**	**Policy makers, service providers**	**Professionals valued relational and informational continuity but managerial continuity (coordination) was limited to own service area**
**Opinion piece**	**[110]**	**USA**	**Presents views on aspects of WCC for USA**	**Evidence**	**Policy makers and providers**	**Offers suggestions that current WCC in US is largely ineffective in addressing long term outcomes**<br><br>**Newer suggested models include:**<br><br>***One-Stop Shopping approach*- other professionals in one stop shop such as social workers etc.**<br><br>***Community Connections approach*-integrated services may be provided at multiple locations with paediatricians as a**

						**coordinator**<br><br>***Paediatricians as medical specialists:*** **WC centres run by public health nurses/educational specialist**
**Literature review**	**[111]**	**USA**	**Evidence for early childhood promotion health topics**	**Evidence**	**Policy makers and providers and parents**	**Early Tobacco exposure prevention, unintentional injury, obesity and mental health program are important and generally cost effective, least available evidence for obesity prevention in Preschool children**
**Literature review**	**[112]**	**Sweden**	**Evidence for fathers involvement on developmental outcomes of children**	**Evidence (22 studies demonstrate positive effect of parental programs)**	**Policy makers and program developers, providers**	**Co-habitation with mother and male partner less externalising behaviour, fathers involvement reduce behavioural problems in boys and psychological problems in young girls, enhance cognitive development and reduce delinquency for low SES families**
**Prospective cohort study**	**[113]**	**Australia**	**Describes maternal and child and provider characteristics associations of health service use**	**Parents use of health services in first twelve months of life**	**Providers and policy makers**	**Mean visits by parents included 32 in first year of life, ~10 to GPs and 12 to nurses, several maternal perceptions and factors predicted GP service use, while nurses use was associated with colic and social support**
**Evaluation study**	**[114]**	**Sweden**	**Evaluates use of health services in Swedish 18 months children in CHS system**	**Parents use of CHS system**	**Providers and policy makers**	**Most families utilise services to optimal level, first time parents visit more, developmental milestones and parental education happens less**

						often
Descriptive study	[115]	Canada	Outlines the changes in the 2006 Rourke Baby health record	Personal health record use update for health supervision	Providers and users in Canada	Importance of 18 months check and more writing space, internet based information included, new immunization record
Literature Review	[116]	Ireland	Literature review of food assessment programs in preschool	Evidence to inform Healthy incentive scheme in Irish full day care preschool setting	Policy makers/providers of child care services and managers	Not known , but impact using obesity and overweight in implementation sites
Literature review	[117]	Australia	Literature review in support of a federally funded national 4 year old Healthy Kids check in Australia	Evidence	Policy makers, providers	Most of the components of the HKC are not evidence based
Comparative Evaluation study	[118]	Canada	Investigate the influence of redistributive policies on social environment of early child development in five countries with different political traditions	Evidence for association of government policies such as prenatal care, maternal leave, child health care and early childhood education on child development	Politicians, policy makers, providers	Generous redistributive policies are associated with higher maternal leave allowance and pay and more preventive child health care visits, no influence on immunization rates and breastfeeding
Literature review	[119]	UK	Highlights based on Marmot review the health inequalities for children	Conceptual framework for policy interventions to reduce inequalities	Policy makers and program developers	Give best start to every child<br><br>Paid parental leave<br><br>Intensify home visiting programs<br><br>Support to families<br><br>Programs for transition to school
Evaluation study	[120]	USA	Understanding the claims data at Alabama in USA	Systems , quality measures for preventive visits	Policy makers and providers	Several disparities of quality of preventive service was seen according to family income, rural location and chronic disease status, differences in Children’s health

						**Insurance program in US limits its usefulness for identifying disparities**
**National framework for Universal Child and Family Health Services**	**[121]**	**Australia**	**Enumerates a national program for states to endorse universal access to child and family services**	**Systems, providers, users**	**States, providers and program developers**	**Endorsed by all states with a commitment for improving child and family services using this framework**
**Evaluation study**	**[122]**	**Australia**	**Understands the barriers and enablers of Practice nurses and GPs for the delivery of 4 year old Healthy kids check (HKC)**	**National screening program delivery for early identifications of problems**	**Providers and policy makers**	**Many providers uncertain regarding their capabilities and practicality for HKC, for some professionals catalysed professional development, linkage to welfare services helped in system improvements (recall, reminders), referral systems affect providers motivations**
**Framework**	**[123]**	**Australia**	**Describes a framework for improving team-based models of primary care**	**Australian primary health care system**	**Policy makers, educators, researchers, managers and health professionals**	**Five domains to support team based models, theory, implementation, infrastructure, sustainability and evaluation**
**Theoretical concepts**	**[124]**	**Sweden**	**Describes the working of policy formation in nine case studies in three municipalities planning child health promotion programs**	**Swedish health promotion programs**	**Policy makers, program makers, researchers**	**Application of Policy Streams approach identified policy entrepreneurs resources of hearing, political connections, and sheer persistence as well the concept of policy window**
**Literature review**	**[125]**	**Australia**	**Reviews literature on Early learning programs promoting children's developmental and educational outcomes**	**In the context of Australian early childhood early programs compares to International data**	**Policy makers, researchers**	**Children's literacy at age 4-5, good predictor of achievement in primary school**<br><br>**Children at risk of poorer outcomes benefit from high quality education and care**

						programs<br><br>Quality of programs is a major determinant<br><br>Indigenous programs need culturally relevant community partnerships
**Practice Nurse (PN) Incentive program**	**[126]**	**Australia**	**Enumerates policies to promote recruitment of practice nurses**	**Improve delivery of services at general practices by enhancing PN**	**Providers at Primary health workforce**	**Increasingly practices in Australia are recruiting Practice nurses**
**Literature review**	**[127]**	**Australia**	**Review of literature of early parenting, education and health intervention programs**	**Indigenous children and families in Australia**	**Providers and policy makers**	**Most programs were effective to varying extents**<br><br>**Communities for children, Brighter Future, Family By Family, Invest to Grow, School as community centres, Hey Dad, Lets start Parent child program, HIPPY**<br><br>***Health intervention programs***<br><br>**Public swimming pools,healthy skin program, Treatment of acute otitis media, Asthma education intervention, Fluoridated water supply**
**Evaluation study**	**[128]**	**Baltimore USA**	**Explores screening for basic social needs of families during WCC visits**	**Screening for employment, education, child care, food and housing of African American mothers attending WCC resident clinic**	**Providers and users**	**Parents reported median of two basic social needs, employment 52%, education 34%, child care 19%, food insecurity 16% and housing 10%**
**Government Report**	**[129]**	**Australia**	**Explores issues in the provision of early childhood education in Australia**	**Early childhood education in Australia compared to international literature**	**Policy makers and early childhood providers**	**Two major models of delivery, government and non-government, access variability within states**

**Evaluation study**	**[130]**	**Australia**	**(Australian Early Development Index (AEDI) Indigenous adaptation study**	**Aimed to evaluate AEDI for indigenous community**	**Policy makers, aboriginal leaders and providers**	**AEDI was a valid tool for indigenous children except in the language and cognitive skills domain (35 percentage point difference)**
**Framework**	**[131]**	**USA**	**Framework for childhood health promotion programs**	**Health promoting capabilities of parents and communities**	**Policy makers and providers**	**Suggest various settings Neighbourhood, workplace, place based centres where policies need to take into account the capacities of family and community for health promotion program**
**Literature review**	**[132]**	**USA/multi-country authorship**	**Makes cross country comparisons of early childhood initiatives and policies, explores differences in theoretical basis**	**Services and systems for early childhood initiatives**	**Policy makers and providers**	**Main programs**<br><br>***England*- Sure Start**<br><br>***Canada*- Toronto First Duty, Ontario Best Start**<br><br>***Australia*- String Families in Communities, Victoria Best Start**<br><br>***USA*-Head Start, Early Head start**
**Evaluation study**	**[133]**	**Sweden**	**Analyse activities and policy processes in 25 Swedish Municipalities**	**Policy approaches to understand what makes thing happen within Swedish system for child health promotion activities**	**Policy makers and program makers**	**Faster growing municipalities with more children and adolescents reported fewer safety promoting activities, lower fraction of preschool staff with a university degree, lower number of full-time employed teachers with university degree**<br><br>**Politician and public officials with strong commitment, professional skills and powerful position**

						**in the system have influence on policy**
**Literature review**	**[134]**	**Australia**	**Reviews evidence for effectiveness of parenting support programs for Indigenous families**	**Australian indigenous communities**	**Policy makers, researchers and program developers**	**Parenting programs promote child well being**<br><br>**Improve some outcomes associated with child abuse and neglect such as poor parent-child interactions**<br><br>**May be more effective as universal primary prevention program**
**Government Report**	**[135]**	**Canada**	**Annual report describing early childhood development major activities and accomplishments: 2011/2012**	**Canadian early childhood sector**	**Policy makers and providers and users**	**Healthy start program, BC Healthy Connections Project (BCHCP) and Nurse family partnership (NFP) and a number of other programs on continuum of pregnancy, infancy and early childhood**
**Literature review**	**[136]**	**UK**	**Literature review to understand published literature on health of the Roma people**	**Roma people living within European Union nations**	**Researchers, policy makers and providers**	**Sparse published literature, need for further research for health of Roma people**
**Conceptual framework**	**[137]**	**Australia**	**Framework for Neighbourhood effects influencing early Childhood development**	**Neighbourhood factors for early childhood development**	**Policy makers and providers**	**Five interconnected domains physical, social, service, socio-economic and governance**
**Evaluation of a program**	**[138]**	**UK**	**Assess the impact of Sure Start Local programmes (SSLP) on seven year olds and their families**	**Areas in UK where disadvantaged families live**	**Policy makers and providers**	**Mothers in SSLP areas showed a greater improvement in home learning, decrease in harsh discipline, improvement in life satisfaction**
**Literature review**	**[139]**	**Australia**	**Review of literature for effective sustained home visiting program for vulnerable families and children**	**Disadvantaged families**	**Policy makers, researchers, politicians and providers**	**Evidence regarding the components of home visiting programs is contradictory**<br><br>**A greater number of visits over long period of time**

						targeting at risk families with complex problems<br><br>Not known why some programs more effective than others
Report	[140]	Ireland	Formulates recommendations in the Irish Best health for Children program	Universal child and family services for Ireland	Policy makers, providers and users	21 Recommendations for Ireland Best Health for Children Program
National Framework	[141]	Northern Ireland	Providers framework for Universal child health promotion programme in Northern Ireland	In the context of the changes in the UK Child health promotion programmes, a review of Northern Ireland promotion program is undertaken	Policy makers, providers and users	Provides clear guidelines of the visits and roles for child health promotion activities, home visiting at 10-14 days<br><br>Reviews at 6-8 weeks, 14-16 wks, 3-4 months, 6 mo, 1 year, 15 mo, 2 years, 3 years and 4-41/2 yrs
Conference Report	[142]	New Zealand	Consensus development on the Tamariki Ora , well child care program in New Zealand	Response to improve outcomes of children from New Zealand	Policy makers, providers and users	Consensus on issues of universal child and family services
State Government Policy	[143]	Australia	State policy on maternal and child health in primary health care	Two components universal and targeted	Policy makers, providers and users	Enumerates model of care for universal assessment, coordinated care, home visiting, by maternity and community services<br><br>SAFE start policy identify parents with mental health problems during perinatal period
Evaluation of program	[144]	New Zealand	Evaluation of the Family Start Program	Early intervention program for New Zealand most at-risk families	Policy makers and providers and users	Average length of program 13-15 months, 90% parents in program received parenting information, families with more contact more positive about the programme
Opinion piece	[145]	New Zealand	Discusses inequalities in child health outcomes in New Zealand	Guidance for programs	Policy makers, providers	Uncertainty how to best address inequalities in NZ child health well- being

						statistics
Research protocol	[146]	UK	Promoting Engagement Project for understanding why early interventions do not reach the children who need them the most	120 families refused to engage with the early intervention	Policy makers, providers	Understanding parental perceptions to inform attempts to engage and retain hard-to-reach families
Government Report	[147]	New Zealand	Improving alignment of Family Start, FS (program for vulnerable families) with Tamariki Ora services	Perceptions of FS and Well child staff for improving coordination	Providers and users and policy makers	Clarity of roles and responsibilities, communication, information sharing, co-location of programmes, professional support emerged as important factors promoting coordination
Literature review	[148]	USA	Empirical Evaluation of Healthy Steps Program for Young Children	21 studies , 13 met the inclusion criteria of empirical evaluation.		Effective in preventing negative child and parent outcomes and enhancing positive outcomes. Cost effectiveness not clear, the Healthy Steps Program provides clear benefit through early screening, family-centered care, and evidence-based anticipatory guidance
Qualitative study	[149]	Australia	Inter-sectoral coordination between non-government organisations from the health and allied health, education and community service sectors	Three small rural communities in Tasmania		Need external and internal leadership
Randomized trial	[150]	USA	Early Head Start, a federal program begun in 1995 for low-income pregnant women and families with infants and toddlers	3,001 families in 17 programs. Interviews with primary caregivers, child assessments, and observations of parent-child interactions were completed when children were 3 years old.		Improved cognitive and language development, higher emotional engagement of the parent ,lower in aggressive 41ehaviour.<br><br>Parents were more emotionally supportive,

						**provided more language and learning stimulation, Most effective a mix of home-visiting and center-based services**
**Collaborative quality improvement program**<br><br>**Cohort study**	**[151]**	**USA**	**12-month program to assist practices in implementing improved systems to provide anticipatory guidance and parental education.**<br><br>**Respectively.**	**Pediatric and family practices in Vermont and North Carolina**<br><br>**Eighteen collaborative education practices and 17 comparison practices**		**care delivery systems increased from a mean of 12.9 to 19.4 of 27 in collaborative practices and remained the same in comparison practices**<br><br>**The proportion of children with documented developmental and psychosocial screening among intervention practices increased from 78% to 88% (P < .001) and from 22% to 29% (P = .002),**
**Opinion piece based on literature view**	**[152]**	**Australia**	**Role of nurses**	**It highlights a number of reasons why some children with subtle developmental difficulties are not identified until school age.**		**Relevant to child health nurses are: a changing focus on early childhood health issues; low repeat attendance at child health clinics, the reluctance of parents to seek assistance; the impact of socio-economic status, cultural and ideological influences; the challenges in using complex screening tools; detecting subtle developmental problems; involvement of parents in decision-making; and, the reluctance of both professionals and parents to 'label' children.**
**Cohort study**<br><br>**Cross sectional evaluation**	**[153]**	**Australia**	**Evaluation of screening and diagnostic outcomes of the New South Wales Statewide Eyesight Preschooler Screening (StEPS) program, a state-funded,**	**A cross-sectional evaluation of the StEPS program, in which eligible 4-year-old children were offered a vision screen in local health**		**Of 91 324 eligible 4-year-olds in NSW,**<br>**80 328 (88.0%) were offered screening, and**<br>**65 834 (72.1% of the**

			**universal vision screening program for 4-year-old children.**	**districts in NSW, between 1 July 2010 and 30 June 2011.**		**eligible population) were screened. Of the children who were screened, 3867 (5.9%) scored less than 6/9-2 but better than 6/18 in one or both eyes and were referred. A further 1425 children (2.2%) scored 6/18 or less in one or both eyes and were referred for high-priority assessment. The StEPS program has achieved a high screening participation rate in NSW.**
**Mental health screening within child health surveillance**	**[154]**	**Denmark**	**Introduction of Copenhagen Infant Mental Health Screening (CIMHS) within the general child health surveillance by community health nurses (CHN).**	**The study population of 2973 infants was assessed by CIMHS at age 9-10 months, and later using validated measures.**		**CIMHS problems of sleep, feeding and eating, emotions, attention, communication, and language were associated with an up to fivefold increased risk of child mental disorders across the diagnostic spectrum of ICD-10 diagnoses. The sensitivity at high CIMHS problem scores was 32% and specificity 86%**
**Cluster randomized quality improvement project**	**[155]**	**USA**	**Introduction of instrument based vision screening test**	**12 paediatric practices**		**Increased completed screening among 3- to 5-year-old children from 54% to 89% in the phase 1 group and from 65% to 92% in the phase 2 group. Improvement was most marked among 3-year-old children. Screening was associated with a 15% reduction in referrals to eye care specialists.**
**Quasi-experimental study**	**[156]**	**Netherlands**	**(FCC-JointStart) family centred care within**	**5658 parents of young children up to 18 months**		**Socio-emotional problems in children were identified more 1.44 (0.96; 2.18), Phi = .03), and earlier**

Systematic review	[157]	USA	Systematic review for primary care interventions reporting outcomes on parenting behaviours and child development	48 studies in which 24 interventions		2 reductions in developmental delay<br><br>4 improved cognitive scores, 6 improved behavioural problems
RCT	[158]	Norway	Maternal and child health centres in 10 municipalities in Norway, introduction of Parent held record	309 parents of children in Norway preschool child health surveillance program		well accepted by parents and professionals but it had no effects on collaboration, healthcare utilization, or other measures that could justify the costs of introducing the record into common use
Cochrane Systematic Reviews	[159]		9 RCT trials with 25,000 participants	Money to relatively poor families	Policy makers and users	No equivocal evidence whether financial benefits delivered as an intervention are effective at improving child health or well-being in the short term
Evaluation study	[160]	Sweden	Review of new problems detected at 18 months exam	>6000 children's records in 1994 and 2000		no evidence that health examinations carried out by nurses are of lower quality in detecting health problems
RCT	[161]	USA	RCT, 168 mothers in each arm participating in WECARE trial	Systematic screening for psychosocial problems	Low income families, used by policy makers	WE CARE mothers received ≥1 referral at the index visit (70% vs 8%; adjusted odds ratio [aOR] = 29.6; 95% confidence interval [CI], 14.7–59.6). At the 12-month visit, more WE CARE mothers had enrolled in a new community resource (39% vs 24%; aOR = 2.1; 95% CI, 1.2–3.7
Before and after intervention design	[162]	Sweden	Salut multi -sectoral population based child health promotion	Interactive lectures and group discussion between	Policy makers and multiple users and providers	an increase in the extent to which midwives in

			program in 13 of 15 Swedish municipalities	Antenatal scare, Community health centres, dental services and open pre-schools		antenatal care raised issues related to men's violence against women, 2) an increase in the extent to which several lifestyle topics were raised with parents/clients in child health care and dental services, 3) an increased use of motivational interviewing (MI) and separate 'fathers visits' in child health care 4) improvements in the supply of healthy snacks and beverages in open pre-schools and 5) increased collaboration between sectors
**Opinion piece**	**[163]**	**Israel**	**Alternatives to developmental screening**	**Based on literature**	**Policy makers**	**Suggested intervention programs for targeted vulnerable populations rather than screening**
**Systematic review and Meta-analysis**	**[164]**	**Multiple sources**	**Incredible year program for any parent with concerns about their child behaviour**	**Meta-analysis**		**Mean effect size d=0.27 for improvement in behaviour**
**Systematic review**	**[77]**	**Multiple sources**	**Triple P parenting program**			**Parenting (overall ES = 0.38), Child Problems (overall ES = 0.35), and Parental Well-Being (overall<br><br>ES = 0.17)**
**Systematic review and Meta-analysis**	**[165]**	**Multiple sources**	**Parenting education for injury prevention**	**Nine RCTs**		**18% mean risk reduction (95% CI 5-29%)**
**Realist review**	**[166]**	**Multiple sources**	**Identifying programme theories of impact of outcomes data on integration and reasons for poor care**	**Synthesis**		**Patient reported outcomes do not give reasons for poor care, three theories patient choice theory, provider benchmarking theory, accountability theory**
**Realist review**	**[167]**		**Impact of one to one breastfeeding supports**	**Synthesis**		**(a) congruence with local infant feeding norms, (b)**

						**integration with the existing system of health care, (c) overcoming practical and emotional barriers to access, (d) ensuring friendly, competent, and proactive peers, (e) facilitating authentic peer-mother interactions, (f) motivating peers to ensure positive within-intervention amplification, and (g) ensuring positive legacy and maintenance of gains**
**Realist review**	**[168]**	**Multiple sources**		**Systematic review**		**This review only identified US research that focused on 'Well Child Care'.**<br><br>**•Found inconclusive evidence in favour of multi-component interventions, QI interventions supported by regulatory change enable more preventive healthcare.,Further research needs to include clinical end points**
**Systematic review**	**[169, 170]**	**Multiple sources**	**Education, interviewing, early identification and documentation in clinics and child care settings**	**Systematic review**		**Change but Little sustained change in BMI scores,**

Table 6 National level indicators which reflect population coverage and success of WCC programs

Country**	Proportion of babies' breast fed up to 6 months exclusive (%)	Proportion of mothers identified with postpartum depression	Proportion of boys (<20 yrs.) identified overweight+ and obese	Proportion of children with developmental vulnerabilities in at least one domain at school	Proportion of babies immunized fully 12 to 23 months	Continuity of provider for well child care (usual source primary care provider)	Proportion of children with ASD (2-17 years), most recent estimates	Proportion watching TV >1 hr <4 hrs (1-17years)	Developmental screening completed (10 months-5years)	Family involved in home visitation program	Proportion of children <5 yrs. visiting dental worker	Proportion of 4year old children enrolled in an early childhood program	Annual number of deaths and injuries 1-14 yrs. per 100000(1991-1995).	Child maltreatment deaths per 100000 children (up to 15yrs)
**USA**	14.8	~16%	28.8	26%	93.7	46	1.8-2.2	40%	30.8%	13.6%	~45$$	65	14.1	2.4
**Australia**	15	15.7-17.4	24	23.6%	92.7	39	1.5-2	40%	N/A$	N/A	~50-60%	64	9.5	0.8
**Canada**	14.4	~15	25.5	25-31%	92.3	50-60	1.1	N/A	N/A$	N/A		-	9.7	1.0
**New Zealand**	19-21	~16	29.6	15-20%	86.7	N/A	1.9	49.8	50-75%$	N/A	59.3	95	13.7	1.3
**Sweden**	10.4	~8-12	20.4	~20%	97.3	N/A	0.5-0.7	N/A	>90%	N/A		90	5.2	0.6
**UK**	~15	10-15	26.1	29%	88	50-68	0.8-1	37	N/A	N/A	N/A	93	6.1	0.9
**Denmark**	~30%		19.7	17%	93.7	N/A		N/A	>90%	N/A	N/A	95	8.1	0.8
**Netherlands**	18%		18.3		97.3	N.A	N/A	N/A	>90%	N/A	N/A	70	6.6	0.6

**All data presented here is best approximate data available after extensive search from multiple references which are enumerated in S1 Appendix. The table above do not highlight regional variations, N/A-comparable data not available, $ Screening conducted at school entry using early development census tool in Canada and Australia; In New Zealand-, New Zealand Before School Check, B4SC, $$ data included children up to 18 years

1. O’Connor ME, Matthews BS, Gao D. Effect of open access scheduling on missed appointments, immunizations, and continuity of care for infant well-child care visits. Archives of Pediatrics & Adolescent Medicine. 2006;160(9):889-93.

2. Chatterji P, Brooks-Gunn J. WIC participation, breastfeeding practices, and well-child care among unmarried, low-income mothers. American Journal of Public Health. 2004;94(8):1324.

3. Chamberlin RW, Szumowski EK. A follow-up study of parent education in pediatric office practices: impact at age two and a half. American Journal of Public Health. 1980;70(11):1180-8.

4. Cassedy A, Fairbrother G, Newacheck PW. The impact of insurance instability on children's access, utilization, and satisfaction with health care. Ambulatory Pediatrics. 2008;8(5):321-8.

5. Cadman D, Chambers LW, Walter SD, Ferguson R, Johnston N, McNamee J. Evaluation of public health preschool child developmental screening: the process and outcomes of a community program. American journal of public health. 1987;77(1):45-51.

6. Brinkman SA, Gialamas A, Rahman A, Mittinty MN, Gregory TA, Silburn S, et al. Jurisdictional, socioeconomic and gender inequalities in child health and development: analysis of a national census of 5-year-olds in Australia. BMJ open. 2012;2(5).

7. Brousseau DC, Meurer JR, Isenberg ML, Kuhn EM, Gorelick MH. Association between infant continuity of care and pediatric emergency department utilization. Pediatrics. 2004;113(4):738-41.

8. Jenkins G, Collins C, Andren S. Developmental surveillance in general practice. British medical journal. 1978;1(6126):1537.

9. Bergman DA, Beck A, Rahm AK. The use of internet-based technology to tailor well-child care encounters. Pediatrics. 2009;124(1):e37-e43.

10. Bornstein MH, Cote LR. “Who Is Sitting Across From Me?” Immigrant Mothers’ Knowledge of Parenting and Children’s Development. Pediatrics. 2004;114(5):e557-e64.

11. Bethell C, Reuland C, Schor E, Abrahms M, Halfon N. Rates of parent-centered developmental screening: disparities and links to services access. Pediatrics. 2011;128(1):146-55.

12. Flocke SA, Litaker D. Physician practice patterns and variation in the delivery of preventive services. Journal of general internal medicine. 2007;22(2):191-6.

13. Tanner JL, Stein MT, Olson LM, Frintner MP, Radecki L. Reflections on well-child care practice: a national study of pediatric clinicians. Pediatrics. 2009;124(3):849-57.

14. Tebruegge M, Nandini V, Ritchie J. Does routine child health surveillance contribute to the early detection of children with pervasive developmental disorders?–An epidemiological study in Kent, UK. BMC pediatrics. 2004;4(1):4.

15. Tough SC, Siever JE, Leew S, Johnston DW, Benzies K, Clark D. Maternal mental health predicts risk of developmental problems at 3 years of age: follow up of a community based trial. BMC pregnancy and childbirth. 2008;8(1):16.

16. Bergman DA, Plsek PE, Saunders M, Fund C. A high-performing system for well-child care: a vision for the future: Commonwealth Fund; 2006.

17. Daubney MF, Cameron CM, Scuffham PA. Changes to the Healthy Kids Check: will we get it right? Medical Journal of Australia. 2013;198:1-3.

18. Russell L. Mental Health provisions in the 2011-2012 budget. 2011, Menzies Health Policy centre, University of Sydney.

19. Walsh JM, McPhee SJ. A systems model of clinical preventive care: an analysis of factors influencing patient and physician. Health Education & Behavior. 1992;19(2):157-75.

20. Wong S, Lynam M, Khan K, Scott L, Loock C. The social paediatrics initiative: a RICHER model of primary health care for at risk children and their families. BMC pediatrics. 2012;12(1):158.

21. Rydz D, Srour M, Oskoui M, Marget N, Shiller M, Birnbaum R, et al. Screening for developmental delay in the setting of a community pediatric clinic: a prospective assessment of parent-report questionnaires. Pediatrics. 2006;118(4):e1178-e86.

22. Ryan-Krause P, Meadows-Oliver M, Sadler L, Swartz MK. Developmental status of children of teen mothers: contrasting objective assessments with maternal reports. Journal of pediatric health care: official publication of National Association of Pediatric Nurse Associates & Practitioners. 2009;23(5):303.

23. Birtwhistle R, Pottie K, Shaw E, Dickinson JA, Brauer P, Fortin M, et al. Canadian Task Force on Preventive Health Care We're back! Canadian Family Physician. 2012;58(1):13-5.

24. McLearn KT, Strobino DM, Hughart N, Minkovitz CS, Scharfstein D, Marks E, et al. Developmental services in primary care for low-income children: clinicians' perceptions of the Healthy Steps for Young Children program. Journal of Urban Health. 2004;81(2):206-21.

25. Riemer JG, Cleve LV, Galbraith M. Barriers to well child care for homeless children under age 13. Public Health Nursing. 2007;12(1):61-6.

26. Rourke L, Leduc D, Constantin E, Carsley S, Rourke J. Update on well-baby and well-child care from 0 to 5 years What's new in the Rourke Baby Record? Canadian Family Physician. 2010;56(12):1285-90.

27. Williams R, Clinton J, Force EYT. Getting it right at 18 months: In support of an enhanced well-baby visit. Paediatrics & child health. 2011;16(10):647.

28. Rosenthal MS, Lannon CM, Stuart JM, Brown L, Miller WC, Margolis PA. A randomized trial of practice-based education to improve delivery systems for anticipatory guidance. Archives of pediatrics & adolescent medicine. 2005;159(5):456-63.

29. Ronsaville D, Hakim R. Well child care in the United States: racial differences in compliance with guidelines. American Journal of Public Health. 2000;90(9):1436.

30. Blair M. The need for and the role of a coordinator in child health surveillance/promotion. Archives of disease in childhood. 2001;84(1):1-5.

31. Roberts G, Efron D, Price A, Hiscock H, Wake M. The Time and Practice Challenges of Developmental-Behavioral Pediatrics: An Australian National Study. Journal of Developmental & Behavioral Pediatrics. 2011;32(5):368-74.

32. Byrd RS, Hoekelman RA, Auinger P. Adherence to AAP guidelines for well-child care under managed care. Pediatrics. 1999;104(3):536-40.

33. Courtney R, Rice C. Investigation of Nurse Practitioner-Patient Interactions: Using the Nurse Practitioner Rating From. The Nurse Practitioner. 1997;22(2):46-65.

34. NSW Ministry of Health. Centre for Epidemiology and Evidence. 2009-2010 Summary Report from the New South Wales Child Health Survey. Sydney. 2012.

35. Regalado M, Halfon N. Primary care services promoting optimal child development from birth to age 3 years: review of the literature. Archives of pediatrics & adolescent medicine. 2001;155(12):1311.

36. Coghlan D, Kiing J, Wake M. Parents' Evaluation of Developmental Status in the Australian day-care setting: Developmental concerns of parents and carers. Journal of paediatrics and child health. 2003;39(1):49-54.

37. Woolfenden S, Eapen V, Williams K, Hayen A, Spencer N, Kemp L. A systematic review of the prevalence of parental concerns measured by the Parents' Evaluation of Developmental Status (PEDS) indicating developmental risk. BMC pediatrics. 2014;14(1):231.

38. Campbell JR, Szilagyi PG, Rodewald LE, Doane C, Roghmann KJ. Patient-specific reminder letters and pediatric well-child-care show rates. Clinical pediatrics. 1994;33(5):268-72.

39. Ou L, Chen J, Hillman K, Eastwood J. The comparison of health status and health services utilisation between Indigenous and non-Indigenous infants in Australia. Australian and New Zealand journal of public health. 2010;34(1):50-6.

40. Ou L, Chen J, Hillman K. Health services utilisation disparities between English speaking and non-English speaking background Australian infants. BMC public health. 2010;10(1):182.

41. Nelson CS, Wissow LS, Cheng TL. Effectiveness of anticipatory guidance: recent developments. Current opinion in pediatrics. 2003;15(6):630-5.

42. Russell LM, Rubin GL, Leeder SR. Preventive health reform: what does it mean for public health? Medical Journal of Australia. 2008;188(12):715.

43. Coker TR, Windon A, Moreno C, Schuster MA, Chung PJ. Well-Child Care Clinical Practice Redesign for Young Children: A Systematic Review of Strategies and Tools. Pediatrics. 2013;131(Supplement 1):S5-S25.

44. Sanghavi DM. Taking well-child care into the 21st century: a novel, effective method for improving parent knowledge using computerized tutorials. Archives of pediatrics & adolescent medicine. 2005;159(5):482.

45. Sand N, Silverstein M, Glascoe FP, Gupta VB, Tonniges TP, O'Connor KG. Pediatricians' reported practices regarding developmental screening: do guidelines work? Do they help? Pediatrics. 2005;116(1):174-9.

46. Kim SS, Frimpong JA, Rivers PA, Kronenfeld JJ. Effects of maternal and provider characteristics on up-to-date immunization status of children aged 19 to 35 months. Journal Information. 2007;97(2).

47. Schmied V, Mills A, Kruske S, Kemp L, Fowler C, Homer C. The nature and impact of collaboration and integrated service delivery for pregnant women, children and families. Journal of Clinical Nursing. 2010;19(23-24):3516-26.

48. Melhuish E, Belsky J, Leyland AH, Barnes J. Effects of fully-established Sure Start Local Programmes on 3-year-old children and their families living in England: a quasi-experimental observational study. The Lancet. 2008;372(9650):1641-7.

49. Melhuish E, Belsky J, Barnes J. Evaluation and value of Sure Start. Archives of disease in childhood. 2010;95(3):159-61.

50. Raban B, Nolan A, Semple C, Dunt D, Kelaher M, Feldman P. Statewide evaluation of best start: Final report. In: University of Melbourne, editor. Melbourne: Department of Human Services; 2006.

51. Valentine K, Katz I, Griffiths M. Early childhood services: models of integration and collaboration. In:UNSW, editor. Perth: Australian Research Alliance for Children and Youth; 2007.

52. The Victorian Government Department of Education and Early Childhood Development: Child Health record: Literature review, Centre for Community Child Health Murdoch Childrens Research Institute. 2012.

53. Bellman M, Vijeratnam S. From child health surveillance to child health promotion, and onwards: a tale of babies and bathwater. Archives of disease in childhood. 2012;97(1):73-7.

54. Barkin SL, Finch SA, Ip EH, Scheindlin B, Craig JA, Steffes J, et al. Is office-based counseling about media use, timeouts, and firearm storage effective? Results from a cluster-randomized, controlled trial. Pediatrics. 2008;122(1):e15-e25.

55. Barkin SL, Scheindlin B, Brown C, Ip E, Finch S, Wasserman RC. Anticipatory guidance topics: are more better? Ambulatory Pediatrics. 2005;5(6):372-6.

56. Bethell C, Peck C, Schor E. Assessing health system provision of well-child care: the Promoting Healthy Development Survey. Pediatrics. 2001;107(5):1084-94.

57. Schuster MA, Duan N, Regalado M, Klein DJ. Anticipatory guidance: what information do parents receive? What information do they want? Archives of pediatrics & adolescent medicine. 2000;154(12):1191-8.

58. Duggan AK, Starfield B, DeAngelis C. Structured encounter form: the impact on provider performance and recording of well-child care. Pediatrics. 1990;85(1):104-13.

59. Berlin A, Hylander I, Törnkvist L. Primary Child Health Care Nurses' assessment of health risks in children of foreign origin and their parents–a theoretical model. Scandinavian journal of caring sciences. 2008;22(1):118-27.

60. Burnip R, Erickson R, Barr GD, Shinefield H, Schoen EJ. Well-child care by pediatric nurse practitioners in a large group practice: a controlled study in 1,152 preschool children. Archives of Pediatrics & Adolescent Medicine. 1976;130(1):51.

61. Glickman M, Gillam S, Boyle G, Woodroffe C. What makes general practitioners do child health surveillance? Archives of disease in childhood. 1994;70(1):47-50.

62. Schmied V, Fowler C, Rossiter C, Homer C, Kruske S. Nature and frequency of services provided by child and family health nurses in Australia: results of a national survey. Australian Health Review. 2014;38(2):177-85.

63. Pittard III WB. Well-child care in infancy and healthcare services utilization from birth to 6 years by late preterm children receiving Medicaid benefits. Southern medical journal. 2013;106(2):173-9.

64. Foley D, Goldfield S, McLoughlim J, Nagorcka J, Oberklaid F, Wake M, et al. A Review of The Early Childhood Literature. Melbourne: The Centre for Community Child Health; 2000. p. 1-36.

65. Boocock SS. Early childhood programs in other nations: goals and outcomes. The Future of Children. 1995:94-114.

66. Barnett WS. Long-term effects of early childhood programs on cognitive and school outcomes. The Future of Children. 1995:25-50.

67. Dworkin P. Preventive health care and anticipatory guidance. Handbook of Early Childhood Intervention. 2nd ed. Cambridge, UK: Cambridge University Press; 2000. p. 327-38.

68. Berkule-Silberman SB, Dreyer BP, Huberman HS, Klass PE, Mendelsohn AL. Sources of parenting information in Low SES Mothers. Clinical pediatrics. 2010;49(6):560-8.

69. Skovgaard AM, Olsen EM, Christiansen E, Houmann T, Landorph S, Jørgensen T. Predictors (0–10 months) of psychopathology at age 1½ years–a general population study in The Copenhagen Child Cohort CCC 2000*. Journal of Child Psychology and Psychiatry. 2008;49(5):553-62.

70. Reijneveld SA, Brugman E, Verhulst FC, Verloove-Vanhorick SP. Identification and management of psychosocial problems among toddlers in Dutch preventive child health care. Archives of pediatrics & adolescent medicine. 2004;158(8):811.

71. Sices L, Feudtner C, McLaughlin J, Drotar D, Williams M. How do primary care physicians identify young children with developmental delays? A national survey. Journal of Developmental & Behavioral Pediatrics. 2003;24(6):409.

72. Schor EL. Rethinking well-child care. Pediatrics. 2004;114(1):210-6.

73. Schempf AH, Minkovitz CS, Strobino DM, Guyer B. Parental satisfaction with early pediatric care and immunization of young children: the mediating role of age-appropriate well-child care utilization. Archives of pediatrics & adolescent medicine. 2007;161(1):50.

74. van Esso D, del Torso S, Hadjipanayis A, Biver A, Jaeger-Roman E, Wettergren B, et al. Paediatric primary care in Europe: variation between countries. Archives of disease in childhood. 2010;95(10):791-5.

75. Søndergaard G, Biering-Sørensen S, Ishøy Michelsen S, Schnor O, Nybo Andersen A-M. Non-participation in preventive child health examinations at the general practitioner in Denmark: A register-based study. Scandinavian journal of primary health care. 2008;26(1):5-11.

76. Freed GL, Spike NA, Sewell JR, Moran LM, Britt H, Valenti L, et al. Changes in longer consultations for children in general practice. Journal of paediatrics and child health. 2013;49(4):325-9.

77. Nowak C, Heinrichs N. A comprehensive meta-analysis of Triple P-Positive Parenting Program using hierarchical linear modeling: Effectiveness and moderating variables. Clinical child and family psychology review. 2008;11(3):114.

78. Kornfält R. Survey of the pre-school child health surveillance programme in Sweden. Acta Paediatrica. 2000;89:2-7.

79. Weber P, Jenni O. Screening in child health: studies of the efficacy and relevance of preventive care practices. Deutsches Ärzteblatt International. 2012;109(24):431.

80. Panjwani S. Parenting interventions to promote child health. Paediatrics and Child Health. 2014;24(3):110-4.

81. Wolfe I, Thompson M, Gill P, Tamburlini G, Blair M, van den Bruel A, et al. Health services for children in western Europe. The Lancet. 2013;381(9873):1224-34.

82. Blair M, Macaulay C. The Healthy Child Programme: how did we get here and where should we go? Paediatrics & Child Health. 2013;24(3):118-23.

83. Blair M, Isaacs A. Evidence-based child helth surveillance for the National child Health Promotion Programme. Current Paediatrics. 2003;13:308-18.

84. Wood R, Blair M. A comparison of Child Health Programmes recommended for preschool children in selected high-income countries. Child: care, health and development. 2013.

85. Ritzwoller DP, Sukhanova A, Beck AL, Bergman D. A new model of well-child care: implications for resource costs and dissemination. The Permanente Journal. 2011;15(2):15.

86. Bremberg S. A perfect 10: Why Sweden comes out on top in early child development programming. Paediatrics & child health. 2009;14(10):677.

87. Fergusson D, Horwood J, Ridder E, Grant H. Early Start Evaluation Report. Christchurch: Christchurch School of Medicine and Health Sciences, Medicine DoP; 2005.

88. Sawyer A, Gialamas A, Pearce A, Sawyer M, Lynch J. Five by Five A Supporting Systems Framework for Child Health and Development. Adelaide: University of Adelaide, Health SoP; 2014.

89. Marmot M, Atkinson T, Bell J, Black C, Broadfoot P, Cumberlege J, et al. Fair Society Healthy Lives- The Marmot Review, Strategic Review of Health Inequalities in England post 2010. In: Statistics OfN, editor. London2010.

90. Kruske S, Schmied V, Sutton I, O'Hare J. Mothers' experiences of facilitated peer support groups and individual child health nursing support: a comparative evaluation. The Journal of perinatal education. 2004;13(3):31.

91. Katz M, Rubino A, Collier J, Rosen J, Ehrich JH. Demography of pediatric primary care in Europe: delivery of care and training. Pediatrics. 2002;109(5):788-96.

92. Pillas D, Marmot M, Naicker K, Goldblatt P, Morrison J, Pikhart H. Social inequalities in early childhood health and development: a European-wide systematic review. Pediatric research. 2014.

93. Wolfe I, Cass H, Thompson MJ, Craft A, Peile E, Wiegersma PA, et al. Improving child health services in the UK: insights from Europe and their implications for the NHS reforms. Bmj. 2011;342.

94. Mchugh A-M, Hornbuckle J. Maternal and Child Health Model of Carein the Aborginal Community Controlled Health Sector. Perth: Aboriginal Health Council of Western Australia; 2011.

95. Page C, Reid A, Hoagland E, Brier Leonard S. WellBabies: mothers' perspectives on an innovative model of group well-child care. Family medicine. 2010;42(3):202.

96. StateGovernmentVictoria. Maternal and Vhild Health Service Guidelines. In: Development DoEaEC, editor. Melbourne: Maternal and Child Office for Children and Portfolio Coordination 2011.

97. Wissow LS, Brown J, Fothergill KE, Gadomski A, Hacker K, Salmon P, et al. Universal mental health screening in pediatric primary care: a systematic review. Journal of the American Academy of Child & Adolescent Psychiatry. 2013;52(11):1134-47. e23.

98. Taylor C. Developments in child health surveillance programmes. Community Practitioner. 2005;77(3):101-4.

99. Sivak L, Arney F, Lewig K. A Pilot Exploration of a Family Home Visiting Program for Families of Aboriginal and Torres Strait Islander Children

Report and Recommendations: Perspectives of Parents of Aboriginal Children and Organisational Considerations. In: Australian Centre for Child Protection UoSA, editor. Adelaide: Family Home Visiting Aborginal Research Partnership; 2008.

100. Sellström E, Guldbrandsson K, Bremberg S, Hjern A, Arnoldsson G. Association between childhood community safety interventions and hospital injury records: a multilevel study. Journal of epidemiology and community health. 2003;57(9):724-9.

101. Rigby MJ, Köhler LI, Blair ME, Metchler R. Child Health Indicators for Europe A priority for a caring society. The European Journal of Public Health. 2003;13(suppl 1):38-46.

102. Thomas R, Doust JA, Vasan K, Rajapakse B, McGregor L, Ackermann E, et al. Identified health concerns and changes in management resulting from the Healthy Kids Check in two Queensland practices. The Medical journal of Australia. 2014;201(7):404-8.

103. Drucquer M. Child health services in France--vive la différence. British medical journal (Clinical research ed). 1983;286(6376):1529.

104. Blair M, Hall D. From health surveillance to health promotion: the changing focus in preventive children’s services. Archives of disease in childhood. 2006;91(9):730-5.

105. Nayeb L, Westerlund M. Språk bedömningar på BVC utvecklas positivt. Lakartidningen. 2014;111(CMY4).

106. Nayeb L, Wallby T, Westerlund M, Salameh EK, Sarkadi A. Child healthcare nurses believe that bilingual children show slower language development, simplify screening procedures and delay referrals. Acta Paediatrica. 2014.

107. Winter Md, Balledux M, Mare Jd. A critical evaluation of Dutch preventive child health care. Child: care, health and development. 1997;23(6):437-

108. Psaila K, Schmied V, Fowler C, Kruske S. Discontinuities between maternity and child and family health services: health professional's perceptions. BMC health services research. 2014;14(1):4.

109. Schmied V, Homer C, Fowler C, Psaila K, Barclay L, Wilson I, et al. Implementing a national approach to universal child and family health services in Australia: professionals' views of the challenges and opportunities. Health & social care in the community. 2014.

110. Coker TR, Thomas T, Chung PJ. Does well-child care have a future in pediatrics? Pediatrics. 2013;131(Supplement 2):S149-S59.

111. Guyer B, Ma S, Grason H, Frick KD, Perry DF, Sharkey A, et al. Early childhood health promotion and its life course health consequences. Academic Pediatrics. 2009;9(3):142-9. e71.

112. Sarkadi A, Kristiansson R, Oberklaid F, Bremberg S. Fathers' involvement and children's developmental outcomes: a systematic review of longitudinal studies. Acta Paediatrica. 2008;97(2):153-8.

113. Goldfield S. Utilisation of health services in the first twelve months of life. Melbourne: Melbourne; 2002.

114. Hagelin E, Jackson K, Wikblad K. Utilization of Child Health Services during the first 18 months of life: aspects of health surveillance in Swedish preschool children based on information in health records. Acta Paediatrica. 1998;87(9):996-1002.

115. Leduc D, Rourke L, Rourke J, Constantin E. Health supervision from zero to five years using the 2006 Rourke Baby Record. Paediatrics & child health. 2006;11(8):487.

116. Molloy CJ, Kearney J, Hayes N, Slattery CG, Corish C. Healthy incentive scheme in the Irish full-day-care pre-school setting. Proceedings of the Nutrition Society. 2014;73(01):147-58.

117. Alexander KE, Mazza D. The Healthy Kids Check-is it evidence-based. Med J Aust. 2010;192(4):207-10.

118. van den Heuvel M, Hopkins J, Biscaro A, Srikanthan C, Feller A, Bremberg S, et al. A comparative analysis of early child health and development services and outcomes in countries with different redistributive policies. BMC public health. 2013;13(1):1049.

119. Simkiss D. Inequalities in children's health in the UK. Paediatrics and Child Health. 2014;24(3):103-9.

120. Menachemi N, Blackburn J, Becker DJ, Morrisey MA, Sen B, Caldwell C. Measuring Prevention More Broadly: An Empirical Assessment of CHIPRA Core Measures. Medicare & medicaid research review. 2013;3(3).

121. Schmied V, Kruske S, Barclay L, Fowler C. National Framework for Universal Child and Family Health Services. In: Australian Government DoHaA, editor. 2011.

122. Alexander KE, Brijnath B, Mazza D. Barriers and enablers to delivery of the Healthy Kids Check: an analysis informed by the Theoretical Domains Framework and COM-B model. Implementation Science. 2014;9(1):60.

123. Naccarella L, Greenstock L, Brooks P. A framework to support team-based models of primary care within the Australian health care system. The Medical journal of Australia. 2013;199(5 Suppl):S22-5.

124. Guldbrandsson K, Fossum B. An exploration of the theoretical concepts policy windows and policy entrepreneurs at the Swedish public health arena. Health promotion international. 2009:dap033.

125. Harrison L, Goldfield s, Metcalfe E, Moore T. Early learning programs that promote children's developmental and educational outcomes (Closing The Gap). In: Welfare AIoHaF, editor. 2012.

126. Medicare. Practice Nurse Incentive Program Guidelines. In: Services DoH, editor. 2012.

127. Bowes J, Grace R. Review of early childhood parenting, education and health intervention programs for Indigenous children and families in Australia. In: Welfare AIoHaF, editor. 2014.

128. Garg A, Butz AM, Dworkin PH, Lewis RA, Thompson RE, Serwint JR. Improving the management of family psychosocial problems at low-income children's well-child care visits: the WE CARE Project. Pediatrics. 2007;120(3):547-58.

129. Baxter J, Hand K. Access to Early Childhood Education in Australia. Melbourne: Australian Institute of Family Studies, 2013.

130. Silburn S, Brinkman S, Ferguson-Hill S, Styles I, Walker R, Shepherd C. The Australian Development Index (AEDI) Indigenous Adaptation Study. In: Research CUoTaTIfCH, editor. Perth2009.

131. Mistry KB, Minkovitz CS, Riley AW, Johnson SB, Grason HA, Dubay LC, et al. A new framework for childhood health promotion: the role of policies and programs in building capacity and foundations of early childhood health. American journal of public health. 2012;102(9):1688-96.

132. Halfon N, Russ S, Oberklaid F, Bertrand J, Eisenstadt N. An International Comparison of Early Childhood Initiatives: From Services to Systems. USA: Commonwealth Fund, 2009.

133. Guldbrandsson K. Child health promotion: Analyses of Activities and Policy Processes in 25 Swedish municipalities. Stockholm, Sweden: Institutionen för folkhälsovetenskap/Department of Public Health Sciences; 2005.

134. Mildon R, Polimeni M. Parenting in the early years:effectiveness of parenting support programs for Indigenous families. In: Welfare AIoHaF, editor. 2012.

135. MinistryofChildren&FamilyDevelopment. British Columbia's Early Years Annual Report, Activities and Expenditures on Early Childhood Development and Early Learning and Child Care. British Columbia2011/2012.

136. Hajioff S, McKee M. The health of the Roma people: a review of the published literature. Journal of Epidemiology and Community Health. 2000;54(11):864-9.

137. Goldfeld S, Woolcock G, Katz I, Tanton R, Brinkman S, O'Connor E, et al. Neighbourhood Effects Influencing Early Childhood Development: Conceptual Model and Trial Measurement Methodologies from the Kids in Communities Study. Social Indicators Research. 2014:1-16.

138. Melhuish E, Belsky J, Leyland AH. The Impact of Sure Start Local Programmes on seven year olds and their families. In: Institure for the Study of Children FaSIDfE, editor. Birkbeck: University of London; 2012.

139. McDonald M, Moore T, Goldfield S. Sustained home visiting for vulnerable families and children: A literature review of effective programs. Parkville, Victoria The Royal Children's hospital Centre for Community Child Health, Murdoch Childrens Research Institute ARACY; 2012.

140. DepartmentofHealth&Children. Best Health for Children Revisited, Report from the National Core Child Health Programme Review Group. In: Children DoHa, editor. Dublin, Ireland2005.

141. DHSSPS. Healthy Child, Healthy Future A framework for the Universal Child Health Promotion Programme in Northern Ireland. In: Department of Health SSaPS, editor. Northern Ireland2010.

142. NZMinistryofHealth. A Consensus Development Conference Report on Well Child Care. Wellington, New Zealand: National Advisory Committee on Core Health nd Disability Support Services; 1993.

143. NSWMinistryofHealth. NSW Health/Families NSWSupporting Families Early Package- Maternal and Child Health Primary Health Care In: Health NDo, editor. North Sydney2009.

144. Centre for Child and Family Policy Research AUL. Outcome/Impact evaluation of Family Start. In: Centre for Social Research and Evaluation MoSD, editor. Auckland2005.

145. D'Souza AJ, Turner N, Simmers D, Craig E, Dowell T. Every child to thrive, belong and achieve? Time to reflect and act in New Zealand. Clinical Correspondence. 2012.

146. Cornah D. The Promoting Engagement Project: why is it that early interventions do not reach the children who need them most? Journal of psychiatric and mental health nursing. 2003;10(4):499-500.

147. MinsitryofHealth. Improving Alignment of Family Startand Well Child Tamariki Ora Services. In: Health Mo, editor. New Zealand 2013.

148. Piotrowski CC, Talavera GA, Mayer JA. Healthy Steps: a systematic review of a preventive practice-based model of pediatric care. Journal of Developmental & Behavioral Pediatrics. 2009;30(1):91-103.

149. Johns S. Early childhood service development and intersectoral collaboration in rural Australia. Australian Journal of Primary Health. 2010;16(1):40-6.

150. Love JM, Kisker EE, Ross C, Raikes H, Constantine J, Boller K, et al. The effectiveness of early head start for 3-year-old children and their parents: lessons for policy and programs. Developmental psychology. 2005;41(6):885.

151. Margolis PA, McLearn KT, Earls MF, Duncan P, Rexroad A, Reuland CP, et al. Assisting primary care practices in using office systems to promote early childhood development. Ambulatory Pediatrics. 2008;8(6):383-7.

152. Williams J, Holmes C. Children of the 21st century: Slipping through the net. Contemporary nurse. 2005;18(1-2):57-66.

153. Blows SJ, Murphy EP, Martin FJ, Davies RM. Vision screening in preschoolers: the New South Wales statewide eyesight preschooler screening program. The Medical Journal of Australia. 2014;200(4):222-5.

154. Ammitzbøll J, Thygesen LC, Holstein BE, Andersen A, Skovgaard AM. Predictive validity of a service-setting-based measure to identify infancy mental health problems: a population-based cohort study. European child & adolescent psychiatry. 2017:1-13.

155. Modest JR, Majzoub KM, Moore B, Bhambhani V, McLaughlin SR, Vernacchio L. Implementation of instrument-based vision screening for preschool-age children in primary care. Pediatrics. 2017;140(1):e20163745.

156. Reijneveld SA, Hielkema M, Stewart RE, A dW. The added value of a family-centered approach to optimize infants' social-emotional development: A quasi-experimental study. PLOS One. 2017;12(12):e0187750.

157. Peacock-Chambers E, Ivy K, Bair-Merritt M. Primary care interventions for early childhood development: a systematic review. Pediatrics. 2017;140(6):e20171661.

158. Bjerkeli Grøvdal L, Grimsmo A, Ivar Lund Nilsen T. Parent-held child health records do not improve care: a randomized controlled trial in Norway. Scandinavian journal of primary health care. 2006;24(3):186-90.

159. Lucas P, McIntosh K, Petticrew M, Roberts HM, Shiell A. Financial benefits for child health and well-being in low income or socially disadvantaged families in developed world countries. The Cochrane Library. 2008.

160. Magnusson M, Sundelin C, Westerlund M. Identification of health problems at 18 months of age–a task for physicians or child health nurses? Child: care, health and development. 2006;32(1):47-54.

161. Garg A, Toy S, Tripodis Y, Silverstein M, Freeman E. Addressing social determinants of health at well child care visits: a cluster RCT. Pediatrics. 2015;135(2):e296-e304.

162. Edvardsson K, Ivarsson A, Garvare R, Eurenius E, Lindkvist M, Mogren I, et al. Improving child health promotion practices in multiple sectors–outcomes of the Swedish Salut Programme. BMC Public Health. 2012;12(1):920.

163. Urkin J, Bar-David Y, Porter B. Should we consider alternatives to universal well-child behavioral-developmental screening? Frontiers in pediatrics. 2015;3.

164. Menting AT, de Castro BO, Matthys W. Effectiveness of the Incredible Years parent training to modify disruptive and prosocial child behavior: A meta-analytic review. Clinical Psychology Review. 2013;33(8):901-13.

165. Kendrick D, Barlow J, Hampshire A, Stewart-Brown S, Polnay L. Parenting interventions and the prevention of unintentional injuries in childhood: Systematic review and meta-analysis. Child: care, health and development. 2008;34(5):682-95.

166. Greenhalgh J, Dalkin S, Gibbons E, Wright J, Valderas JM, Meads D, et al. How do aggregated patient-reported outcome measures data stimulate health care improvement? A realist synthesis. Journal of Health Services Research & Policy. 2017:1355819617740925.

167. Trickey H, Thomson G, Grant A, Sanders J, Mann M, Murphy S, et al. A realist review of one-to-one breastfeeding peer support experiments conducted in developed country settings. Maternal & child nutrition. 2017.

168. Alexander KE, Brijnath B, Biezen R, Hampton K, Mazza D. Preventive healthcare for young children: A systematic review of interventions in primary care. Preventive Medicine. 2017.

169. Sargent G, Pilotto L, Baur L. Components of primary care interventions to treat childhood overweight and obesity: a systematic review of effect. Obesity reviews. 2011;12(5).

170. Bluford DA, Sherry B, Scanlon KS. Interventions to prevent or treat obesity in preschool children: a review of evaluated programs. Obesity. 2007;15(6):1356-72.
